# Site-specific ubiquitination of VDAC1 restricts its oligomerization and mitochondrial DNA release in liver fibrosis

**DOI:** 10.1038/s12276-022-00923-9

**Published:** 2023-01-19

**Authors:** Ne N. Wu, Lifeng Wang, Lu Wang, Xihui Xu, Gary D. Lopaschuk, Yingmei Zhang, Jun Ren

**Affiliations:** 1 Department of Cardiology, Shanghai Institute of Cardiovascular Diseases, Zhongshan Hospital Fudan University, Shanghai, 200032 China; 2 National Clinical Research Center for Interventional Medicine, Shanghai, 200032 China; 3 Department of Physiology, School of Basic Medical Sciences, Xinjiang Medical University, Urumqi, Xinjiang 830000 China; 4 Institute of Digestive Diseases, Xijing Hospital, Air Force Medical University, Xi’an, 710032 China; 5 State Key Laboratory of Cancer Biology, Department of Biochemistry and Molecular Biology, Air Force Medical University, Xi’an, 710032 China; 6 Cardiovascular Research Centre, University of Alberta, Edmonton, Alberta T6G 2S2 Canada; 7 Department of Laboratory Medicine and Pathology, University of Washington, Seattle, WA 98195 USA

**Keywords:** Ubiquitylation, Mitochondria

## Abstract

Mitochondrial DNA (mtDNA) released through protein oligomers, such as voltage-dependent anion channel 1 (VDAC1), triggers innate immune activation and thus contributes to liver fibrosis. Here, we investigated the role of Parkin, an important regulator of mitochondria, and its regulation of VDAC1-mediated mtDNA release in liver fibrosis. The circulating mitochondrial DNA (mtDNA) and protein levels of liver Parkin and VDAC1 were upregulated in patients with liver fibrosis. A 4-week CCl_4_ challenge induced release of mtDNA, activation of STING signaling, a decline in autophagy, and apoptosis in mouse livers, and the knockout of Parkin aggravated these effects. In addition, Parkin reduced mtDNA release and prevented VDAC1 oligomerization in a manner dependent on its E3 activity in hepatocytes. We found that site-specific ubiquitination of VDAC1 at lysine 53 by Parkin interrupted VDAC1 oligomerization and prevented mtDNA release into the cytoplasm under stress. The ubiquitination-defective VDAC1 K53R mutant predominantly formed oligomers that resisted suppression by Parkin. Hepatocytes expressing VDAC1 K53R exhibited mtDNA release and thus activated the STING signaling pathway in hepatic stellate cells, and this effect could not be abolished by Parkin. We propose that the ubiquitination of VDAC1 at a specific site by Parkin confers protection against liver fibrosis by interrupting VDAC1 oligomerization and mtDNA release.

## Liver disease: a protein to fight fibrosis

The molecular mechanisms causing liver fibrosis, the tissue scarring associated with long-term liver disease, involve an enzyme called Parkin, suggesting that drugs enhancing Parkin activity might delay, reduce or prevent fibrosis. Researchers in China led by Jun Ren and Yingmei Zhang at Fudan University, Shanghai, studied cells from human and mouse liver to investigate the release of DNA from cell organelles called mitochondria, a key feature in the development of liver fibrosis. This DNA release triggers the abnormal immune activity implicated in causing fibrosis. The researchers uncovered a molecular interaction allowing Parkin to restrict the release of DNA from mitochondria. Parkin achieves this effect by modifying a protein channel in the mitochondrial membrane. Enhancing this enzyme’s protective ability could be explored in search of new therapeutic approaches.

## Introduction

Regardless of the nature of the etiology, it is well perceived that the innate immune response evoked by damage-associated molecular patterns (DAMPs) commonly serves as an initial event in amplifying sterile inflammation to trigger the transition into fibrosis in multiple organ systems, including the liver^1–4^. The mitochondrial content, particularly that of mitochondrial DNA (mtDNA), can be recognized as endogenous DAMPs when released into the cytosol and extracellular compartment due to its bacterial origin^5,6^. Human and animal models of liver fibrosis often display disorganized mitochondria, compromised respiratory function, increased oxidative stress, and elevated release of hepatocyte mitochondria-derived DAMPs, with mtDNA acting as a major active component of DAMPs^3,7,8^. Of note, obese patients with high serum alanine aminotransferase (ALT) often exhibit increased serum levels of mtDNA but not necessarily nuclear DNA^9^. mtDNA released into the cytoplasm instigates the activation of Toll-like receptors (TLRs) and the cGAS (cyclic GMP–AMP synthase)-STING (stimulator of interferon genes protein) signaling axis to induce the expression of various inflammatory cytokines and chemokines^2,10^, which contributes to the onset and development of hepatic fibrosis. Similarly, STING deficiency was found to prevent hepatocyte death and liver fibrosis in both acute and chronic settings of carbon tetrachloride (CCl_4_) challenge in mice^1^.

Several mechanisms in restraining hepatic innate immune activation have been postulated^4^. Mitophagy, the selective engulfment of senescent (long-lived) or damaged mitochondria into autophagosomes that fuse with lysosomes for degradation, is an essential mechanism that prevents the release of mitochondria-associated DAMPs^11^. Mitophagy is mainly mediated by a Parkin-dependent mechanism, in which the E3 ubiquitin ligase Parkin is recruited to depolarized mitochondria to ubiquitinate mitochondrial outer membrane (MOM) proteins and hence tags the removal of long-lived or injured mitochondria^11,12^. Increased levels of circulating mtDNA have been suggested to play an important role in the activation of STING-dependent inflammation in stressed mice lacking Parkin and PINK1^13^. Mitochondrial outer membrane permeabilization (MOMP) is needed for mtDNA release^14,15^. In addition to BAX/BAK oligomers, voltage-dependent anion channel 1 (VDAC1) oligomerizes and forms large MOM pores in the face of oxidative stress^14^. Parkin has been proposed to ubiquitinate a number of MOM proteins to regulate their function and activity, including the state of oligomerization^16^. To this end, the present study was designed to decipher the potential interplay between Parkin and mtDNA release and the possible involvement of VDAC1 oligomers in liver fibrosis. Our results demonstrated that Parkin restricts both mtDNA release into the cytosol and STING activation by ubiquitinating VDAC1 and thus preventing its oligomerization in liver fibrosis.

## Materials and methods

### Human samples

Human liver and serum samples were obtained from healthy subjects and patients with liver fibrosis (*n* = 7 for liver samples, *n* = 12 for serum samples) (patient information presented in Supplementary Table 1 and 2). The protocol involving human subjects received approval (#KY20213504-1) from the Institutional IRB Committee of Xijing Hospital Air Force Military Medical University (Xi’an, China) and adhered to the principles defined in the Declaration of Helsinki.

### Experimental animals

The experimental procedures were approved by the Institutional Animal Use and Care Committee at Zhongshan Hospital Fudan University (Shanghai, China) and complied with the NIH Guide for the Care and Use of Laboratory Animals. In brief, wild-type (WT) and global Parkin-knockout (Parkin^-/-^) mice (B6.129S4-Parktm1Shn/J, strain 006582) on a C57BL/6 J background were obtained from the Jackson Laboratory (Bar Harbor, ME, USA), and the genotypes were confirmed by PCR (primers provided in Supplementary Table 4). Liver fibrosis was established via intraperitoneal injection of CCl_4_ (Sigma‒Aldrich, St. Louis, MO, USA; 1 ml/kg body weight, diluted 1:4 v/v in corn oil) twice per week for 4 weeks^17^. An equal amount of vehicle (corn oil) was delivered to the control mice. The mice were sacrificed by cervical dislocation 3 days following the final CCl_4_ injection under anesthesia with a combination of ketamine (80 mg/kg; Pfizer, Berlin, Germany) and xylazine (12 mg/kg; Bayer AG, Leverkusen, Germany). Blood and liver tissue samples were collected immediately. The serum levels of alanine transaminase (ALT) and aspartate transaminase (AST) were analyzed using the VetACE Clinical Chemistry System (Alfa Wassermann Inc., West Caldwell, NJ, USA)^18^.

### Real-time quantitative polymerase chain reaction (qPCR)

Total RNA of liver tissue was extracted using TRIzol Reagent (Invitrogen, USA). The purity and concentration of RNA were determined using a NanoDrop 2000 spectrophotometer (Thermo Fisher Scientific, Waltham, MA, USA). Reverse transcription was conducted using PrimeScript^TM^ RT Master Mix (Takara Bio Inc., Shiga, Japan) for the synthesis of cDNA. For human serum mtDNA quantification, total DNA was extracted from 200 μl of serum with a Serum/Plasma Circulating DNA Kit (Tiangen, Beijing, China). Real-time qPCR was performed with a QuantiTect SYBR Green PCR kit (Takara Bio Inc., Shiga, Japan) and a CFX Connect Real-Time PCR Detection System (Bio-Rad, Hercules, CA, USA) with the specific primers (Dongxuan Gene, Kunshan, China) listed in Supplementary Table 4. The relative mRNA levels of collagen type I alpha 1 (*Col1a1*), actin alpha-smooth muscle (*Acta22*), and matrix metallopeptidase 2 (*Mmp2*) and the serum mtDNA levels were calculated using the ΔCt method^19^.

### Histological examination

Following anesthesia and cervical dislocation, livers were exercised and placed in 10% neutral-buffered formalin for 24 hours at room temperature. Tissue specimens were embedded in paraffin, sliced into 5-μm sections, and stained with Masson’s trichrome or Sirius red staining. Images were captured with an Olympus BX-51 microscope (Olympus America Inc., Melville, NY, USA) and analyzed using ImageJ Fiji software (version 2.3.0, NIH)^20^.

### Transmission electron microscopy (TEM)

Cubic liver pieces were fixed with 2.5% glutaraldehyde diluted in sodium phosphate (0.1 M, pH 7.4) for 24 h at 4 °C. Tissue samples were dehydrated through a graded alcohol series and embedded in Epon Araldite after postfixation in 1% OsO4 for 1 h. Ultrathin sections (50 nm) were produced using an ultramicrotome (Leica, Wetzlar, Germany) prior to staining with uranyl acetate and lead citrate. The specimens were visualized using an electron microscope. Images were taken using an FEI Tecnai G2 Spirit transmission electron microscope (Hillsboro, OR, USA)^21,22^.

### Determination of NAD^+^

After being processed into powders in a mortar under liquid nitrogen, liver tissues (30 mg) were thoroughly blended with 0.6 M perchloric acid (150 μl) prior to homogenization and neutralization using potassium hydroxide (3 M). Following centrifugation, supernatants were collected to measure the NAD^+^ content using an alcohol dehydrogenase fluorometric assay (Spectra MaxGeminiXS, Sunnyvale, CA, USA) with excitation and emission wavelengths of 339 nm and 460 nm, respectively^23^.

### Aconitase activity

An aconitase-340 assay kit (OxisResearch, Portland, OR, USA) was used to detect NADPH formation during the oxidation of isocitrate to α-ketoglutarate. Mitochondria (50 μl) were thoroughly blended with the substrate (50 μl of trisodium citrate, pH 7.4), enzyme (50 μl of isocitrate dehydrogenase), and NADP^+^ reagent (50 μl). The mixture was cultivated at 37 °C for 15 min, and the absorbance at 340 nm was then measured^23^.

### Evaluation of apoptosis

Terminal deoxynucleotidyl transferase (TdT)-mediated dUTP nick-end labeling (TUNEL) staining was employed to determine hepatic apoptosis using an in situ cell death detection kit (Roche, Basel, Switzerland) per the manufacturer’s guide before 4’,6-diamidino-2-phenylindole (DAPI) staining. Images were generated using an Olympus BX-51 microscope (Olympus America Inc., Melville, NY, USA). The TUNEL-positive nuclei were detected for the determination of apoptosis using ImageJ Fiji software (version 2.3.0, NIH)^18^.

### Bioinformatics analysis

The mRNA profiles of human livers were downloaded from the Gene Expression Omnibus database (GEO: GSE171248)^24^. Genes meeting the criteria of |log_2_Foldchange | >2.0 and *p* value < 0.05 were identified as differentially expressed genes (DEGs) between 8 individuals with histologically normal livers (HNL) and 8 sex- and age-matched patients with liver cirrhosis using the “DESeq2” R package^25^. The R package “clusterprofiler” was utilized to conduct the gene ontology (GO) enrichment analysis^26^. The mRNA levels of 312 common interferon-stimulated genes (ISGs)^27^ (listed in Supplementary Table 3) were screened, and the differentially expressed ISGs are presented in the heatmap.

### Cell culture and transfection

Human hepatoma HepG2 cells and the human hepatic stellate cell line LX-2 (Zhong Qiao Xin Zhou Biotechnology Co., Ltd., Shanghai, China) were cultured in Dulbecco’s Modified Eagle’s Medium containing 10% fetal bovine serum (Thermo Fisher Scientific Inc., Waltham, MA, USA). Small interfering RNA (siRNA) targeting endonuclease (*ENDOG*) (5’-CCGCAGCTTGACGCGAACTTA-3’) and scramble siRNA were obtained from Hanbio Biotechnology Co. (Shanghai, China). The N-terminal tagged WT and mutant gene fragments (Flag-Ubiquitin WT/K6/K11/K27/K29/K33/K48/K63, HA-VDAC1 WT/N-KR/K53R/K274R and Myc-Parkin WT/C315S) were amplified and cloned into the pcDNA3.1(+) vector to construct recombinant plasmids (Dongxuan Genes, Kunshan, China). HepG2 cells were transiently transfected with siRNA or plasmids using Lipofectamine 3000 Reagent (Thermo Fisher Scientific Inc., Waltham, MA, USA) for 48 hours according to the manufacturer’s instruction with or without incubation with DMSO vehicle or Mdivi-1 (50 μM, Sigma‒Aldrich, St. Louis, MO, USA) for 24 h. After 48 h, conditioned media of HepG2 cells were removed and filtered (0.22 micron) prior to the addition to LX-2 cells for another 24 h^28^.

### Ubiquitination assays

Immunoprecipitation (IP) was performed in denaturing RIPA buffer [50 mM Tris-Cl, pH 7.4, 150 mM NaCl, 5 mM EDTA, 1% (v/v) Triton X-100, 0.5% sodium pyrophosphate, 0.1% SDS, and protease inhibitor cocktail (Roche, Basel, Switzerland)]. The cell lysates were centrifuged at 22,500 × *g* and 4 °C for 15 min, and the supernatants were incubated with anti-HA (Shanghai Genomics Inc., Shanghai, China)/VDAC1 beads at 4 °C overnight. The beads were then resuspended, extensively washed three times with RIPA buffer (10 min at 4 °C for each wash), and subjected to SDS‒PAGE and immunoblotting (IB) analysis with anti-Flag (ubiquitin) antibodies^29^.

### Cytosolic mtDNA assessment

Liver tissues and HepG2 cells were homogenized in 100 mM Tricine-NaOH solution containing 0.25 M sucrose, 1 mM EDTA, and protease inhibitor with a pH of 7.4. The samples were then centrifuged at 4 °C and 700 × g for 10 min to remove the nucleus and cellular debris. The supernatant was collected and centrifuged at 10,000 × *g* for 30 minutes at 4 °C to remove mitochondria. DNA was isolated from the whole-cell extracts and cytosolic fractions using a DNeasy Blood & Tissue Kit (Qiagen, Hilden, Germany). The relative mtDNA levels were determined by qPCR with the primers listed in Supplementary Table 4^19^.

Cytosolic mtDNA was also detected by immunofluorescence staining in HepG2 cells. In brief, the cells were transduced with siRNA targeting *ENDOG* and Myc-Parkin plasmids. The cells were stained with MitoTracker (500 nM, Cell Signaling Technology, Danvers, MA, USA) for 30 min at 37 °C, fixed with ice-cold paraformaldehyde for 20 min, permeabilized with 0.5% Triton X-100 for 10 minutes and incubated with PBST containing 5% BSA for 1 h and then with an anti-double-stranded DNA (dsDNA) primary antibody (1:500, #27156, Abcam, Cambridge, England) in a humidified chamber overnight at 4 °C. The cells were incubated with Alexa Fluor 488-conjugated secondary antibody (1: 500, Abcam, Cambridge, England) for 1 h at room temperature prior to DAPI staining. Images were acquired using a confocal microscope (TCS SP8, Leica, Heidelberg, Germany)^30^.

### VDAC1 cross-linking assay

To decipher the oligomeric forms of VDAC1, chemical crosslinking was performed in liver tissues and cells using the membrane permeable cross-linker EGS [ethylene glycol bis (succinimidyl succinate), Thermo Fisher Scientific Inc., Waltham, MA, USA] to stabilize oligomers. Briefly, cells were harvested by scraping and rinsed twice with PBS. Cells or protein lysates of liver tissue were incubated with 0.5 mM EGS in PBS, pH 7.4, at 30 °C for 20 minutes. To remove possible excess cross-linkers, 1.5 M Tris HCl (pH 7.8) was supplied to a final concentration of 20 mM, and the mixture was incubated for 5 minutes at room temperature prior to centrifugation at 10,000 × *g* for 5 minutes. The pellets were lysed in NP-40 lysis buffer by sonication on ice. The protein content was measured using the Pierce BCA Protein Assay (#P0011, Beyotime, Shanghai, China). The samples (50 µg) were then subjected to SDS‒PAGE and immunoblotting using an anti-VDAC1 antibody (#154856, Abcam, Cambridge, UK)^14,31^.

### Western blot analysis

Liver tissues or HepG2 cells were homogenized and sonicated in lysis buffer containing 20 mM Tris (pH 7.4), 150 mM NaCl, 1 mM EDTA, 1 mM EGTA, 1% Triton, 0.1% sodium dodecyl sulfate, and a protease inhibitor cocktail. The samples were incubated with the antibodies listed in Supplementary Table 5. After IB, films were detected with a ChemiDoc Imaging System (Bio-RAD, Hercules, CA, USA) and analyzed using ImageJ Fiji software (version 2.3.0, NIH)^30^.

### Statistical analysis

The data are expressed as the means ± SEMs. The statistical significance (*p < 0.05, **p < 0.01, ***p < 0.001 and **** *p* < 0.0001) was estimated by unpaired t test, one-way or two-way analysis of variance (ANOVA) followed by Tukey’s test for *post hoc* analysis. All statistics were performed with GraphPad Prism 9.3.0 software (GraphPad, San Diego, CA, USA).

## Results

### Parkin knockout aggravates CCl_4_-induced liver fibrosis in mice

**Fig. 1:**
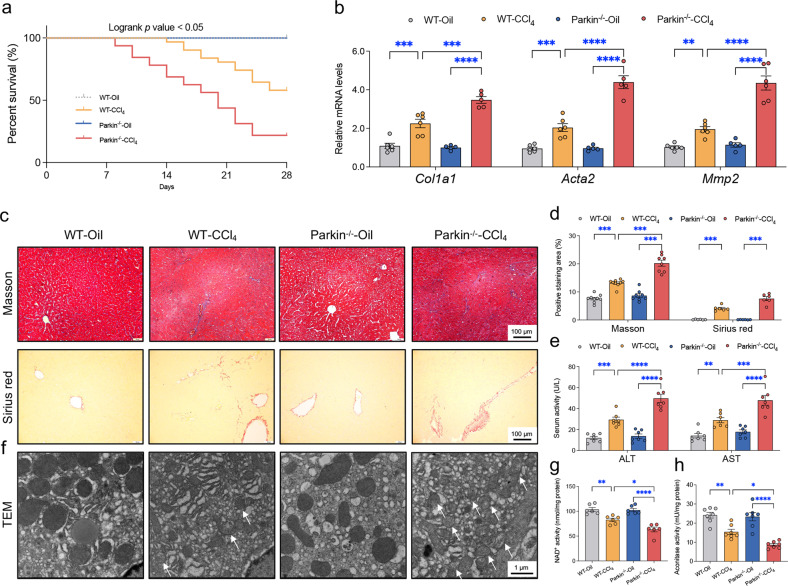
Effects of Parkin knockout on the 4-week CCl_4_ injection-induced serum and histological changes in mice. Blood and liver samples were collected 3 days after the final CCl_4_ injection. (**a**) Survival rate (*n* = 31 mice/group). (**b**) Quantified relative mRNA levels of *Col1a1*, *Acta22*, and *Mmp2* normalized to the *Gapdh* levels in mouse livers (*n* = 5-6/group). (**c**) Representative images of Masson trichrome and Sirius red staining of the liver. (**d**) Quantified fibrotic area based on Masson trichrome staining (*n* = 6/group) and Sirius red staining (*n* = 8-9/group). (**e**) Serum ALT and AST levels (*n* = 7/group). (**f**) Representative TEM images of livers (*n* = 4-5/group). The white arrows denote damaged mitochondria (shrunk mitochondria, disorganized mitochondrial cristae, and broken mitochondrial membrane). (**g**) NAD^+^ level depicting mPTP opening. (**h**) Mitochondrial aconitase activity. Mean ± SEM, *p < 0.05, **p < 0.01, ***p < 0.001 and **** *p* < 0.0001 between the indicated groups.

A cohort of WT and Parkin^-/-^ mice were intraperitoneally injected with CCl_4_ twice per week for 4 weeks to establish experimental liver fibrosis and were checked every other day. Markedly higher mortality was noted in response to the 4-week CCl_4_ injection in the Parkin^-/-^ mice in comparison with the WT mice (Fig. 1a). CCl_4_ injection resulted in liver fibrosis with a more pronounced effect in Parkin^-/-^ mice, as shown by the hepatic mRNA levels of markers for hepatic stellate cell (HSC) activation and fibrosis (*Col1a1*, *Acta22*, and *Mmp2*) (Fig. 1b) as well as Masson and Sirius red staining (Fig. 1c, d). The serum ALT and AST activities were overtly elevated in response to CCl_4_ challenge, and a more pronounced effect was observed in Parkin^-/-^ mice (Fig. 1e). In addition, Parkin ablation substantially accentuated the shrinkage of mitochondria, and more disorganized and fragmented cristae were detected in the face of CCl_4_ challenge (Fig. 1f). Moreover, CCl_4_-induced mitochondrial permeation transient pore (mPTP) opening, as evidenced by decreased NAD^+^ levels (Fig. 1g) and mitochondrial aconitase loss (Fig. 1h), was exacerbated by Parkin ablation. These data indicated that Parkin removal accentuated liver fibrosis and mitochondrial damage evoked by CCl_4._

### The CCl_4_-induced changes in autophagy and apoptosis are augmented by Parkin ablation

**Fig. 2:**
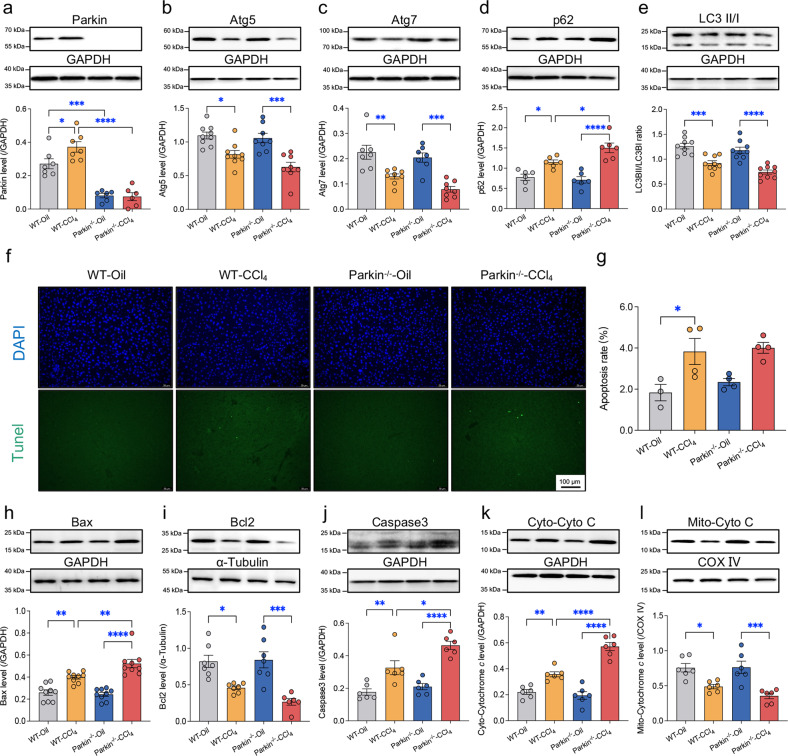
Effects of Parkin ablation on autophagy and apoptosis protein markers in livers following CCl_4_ challenge. Representative immunoblots and quantitative histogram of the autophagy markers (**a**) Parkin, (**b**) Atg5, (**c**) Atg7, (**d**) p62, and (**e**) LC3BII/I and the cell death-related proteins (**h**) Bax, (**i**) Bcl2, (**j**) Caspase3, (**k**) cytosolic Cytochrome C, and (**l**) mitochondrial Cytochrome C in the liver tissues from WT and Parkin^-/-^ mice injected with CCl_4_ or oil for 4 weeks. GAPDH, α-tubulin or COX IV was used as the loading control (*n* = 6-9/group). (**f**) Representative DAPI/TUNEL staining images of mouse livers. (**g**) Quantified TUNEL-positive nuclei (*n* = 4-5/group). Mean ± SEM, *p < 0.05, **p < 0.01, ***p < 0.001 and **** *p* < 0.0001 between the indicated groups.

Our data revealed that Parkin was induced by CCl_4_ in murine livers (Fig. 2a). However, CCl_4_ injection suppressed autophagy, and this suppression was exaggerated by Parkin deletion, as manifested by decreased levels of Atg5 and Atg7 and the LC3BII-to-LC3BI ratio along with increased p62 levels (Fig. 2b–e). Autophagy/mitophagy is a critical machinery through which damaged mitochondria are removed to prevent cell damage and death^32^. We noted increased TUNEL-positive staining (Fig. 2f–g), upregulated Bax and Caspase3 expression and downregulated Bcl2 expression in mouse livers following CCl_4_ injection, and a more profound response was observed in Parkin^-/-^ mice than in WT mice (Fig. 2h–j). Parkin deletion exacerbated the CCl_4_-induced translocation of cytochrome C from mitochondria into the cytosol, indicating that MOMP formation led to cell death (Fig. 2k–l). Thus, loss of Parkin suppressed autophagy and deteriorated mitochondria-mediated cell death following CCl_4_ challenge.

### The circulating mtDNA levels and hepatic STING signaling are elevated in patients with liver fibrosis

**Fig. 3:**
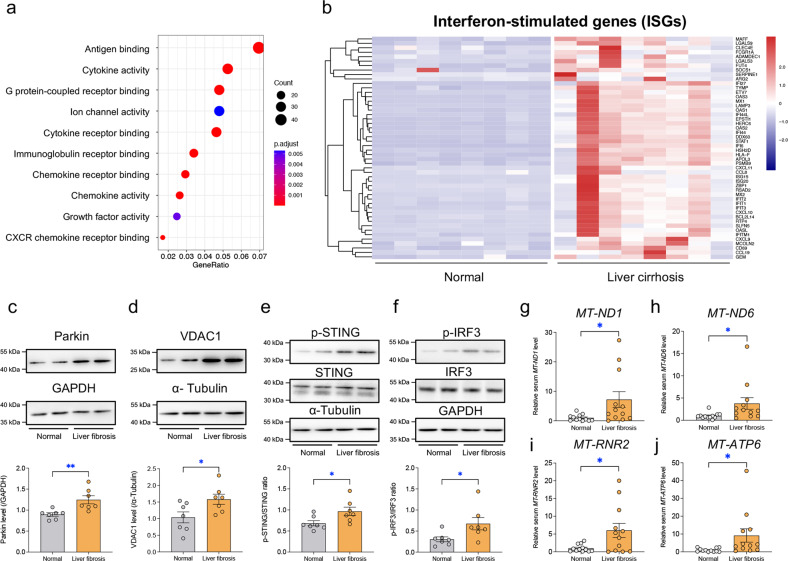
Circulation mtDNA and protein expression in human serum and liver tissues. **a** Top 10 enriched GO molecular functions in livers from healthy subjects and patients with liver cirrhosis. **b** Heatmap presenting differentially expressed interferon-stimulated genes (ISGs) between human cirrhotic and normal livers. Representative immunoblots and quantitative histogram of (**c**) Parkin, (**d**) VDAC1, (**e**) p-STING/STING, and (**f**) p-IRF3/IRF3 in human liver tissues (GAPDH or α-Tubulin was used as the loading control) (*n* = 7/group). Quantified relative levels of the mtDNA reference genes (**g**) *MT-ND1*, (**h**) *MT-ND6*, (**i**) *MT-RNR2*, and (**j**) *MT-ATP6* normalized to the nuclear reference gene β-2 microglobulin in human serum (*n* = 12/group). Mean ± SEM, *p < 0.05 and ** *p* < 0.01 between the indicated groups.

To discern the underlying mechanism in human liver fibrosis, the publicly available RNA sequencing (RNA-seq) results (GSE171248) of livers from 8 liver cirrhotic patients and 8 subjects with histologically normal livers were downloaded from the Gene Expression Omnibus (GEO) database^24^. A GO enrichment analysis revealed that the majority of enriched molecular functions were related to activation of the immune response, such as cytokine activity, chemokine receptor binding, and antigen binding (Fig. 3a). Thus, we screened the levels of 312 common ISGs^27^ in these RNA-seq data, and 56 of these ISGs were significantly changed (Fig. 3b).

STING is an important signal transducer in innate immune signaling that amplifies the induction of type-I interferon (IFN-I) in response to cytosolic DNA, including mtDNA released through VDAC1 oligomers^14^. Therefore, the expression or activation levels of Parkin, VDAC1, STING, and IRF3 were monitored in human liver samples. Elevated levels of Parkin and VDAC1 along with higher levels of p-STING and p-IRF3 were identified in fibrotic livers compared with normal livers (Fig. 3c–f). Furthermore, the circulating mtDNA levels were examined using the serum from human subjects. The serum mtDNA levels (evaluated using 4 mitochondrial genes) were increased in patients clinically diagnosed with liver fibrosis compared with otherwise healthy subjects (Fig. 3g–j). These data suggested that liver fibrosis was closely associated with the activation of the STING signaling pathway induced by mtDNA release in human subjects.

### Parkin ablation exacerbates CCl_4_-induced hepatic mtDNA release and signaling events downstream of STING

**Fig. 4:**
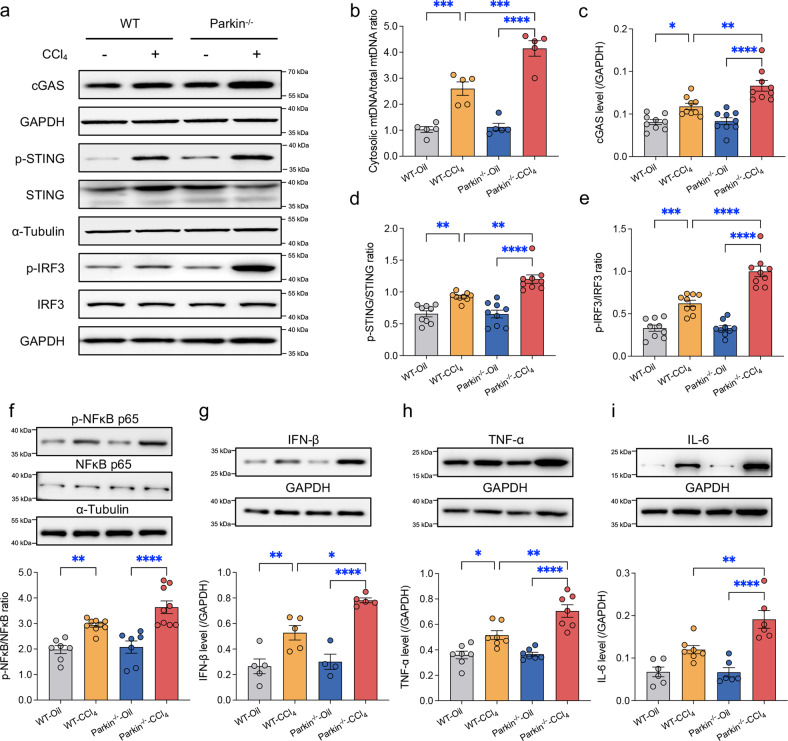
Effects of Parkin knockout on mtDNA release and activation of STING signaling. **a** Representative immunoblots of cGAS, p-STING/STING, and p-IRF3/IRF3 in WT and Parkin^-/-^ mouse livers 4 weeks after injection of CCl_4_ or oil (tissues were collected 3 days after the final injection). **b** Relative ratio of cytosolic to total mtDNA (mt-Nd1) in mouse livers (*n* = 5/group). Quantitative histogram of (**c**) cGAS, (**d**) p-STING/STING, and (**e**) p-IRF3/IRF3 expression levels in the mouse liver. Representative immunoblots and quantitative histogram of (**f**) p-NFκB p65/NFκB p65, (**g**) IFN-β, (**h**) TNF-α, and (**i**) IL-6 (GAPDH or α-Tubulin used as the loading control) (*n* = 5-9/group). Mean ± SEM, *p < 0.05, **p < 0.01, ***p < 0.001 and **** *p* < 0.0001 between the indicated groups.

We subsequently explored whether the effects of Parkin in liver fibrosis are mediated by mtDNA-STING-dependent proinflammatory signaling. Parkin^-/-^ mice exhibited increased production of cGAS and higher phosphorylation states of STING, IRF3, and NFκB p65 than WT mice following CCl_4_ injection (Fig. 4a, c–f), indicating an inhibitory effect of Parkin against STING-mediated proinflammatory responses in the liver. Consistently, CCl_4_ challenge resulted in markedly higher cytosolic mtDNA levels (Fig. 4b) and upregulated levels of IFN-β, TNF-α and IL-6 (Fig. 4g–i) in the livers of Parkin^-/-^ mice compared with WT mice. These data suggested that the CCl_4_-induced activation of STING signaling was amplified by Parkin knockout, prompting the notion of Parkin-regulated mtDNA release in the liver.

### Parkin knockout promotes oligomerization of VDAC1 in the liver

**Fig. 5:**
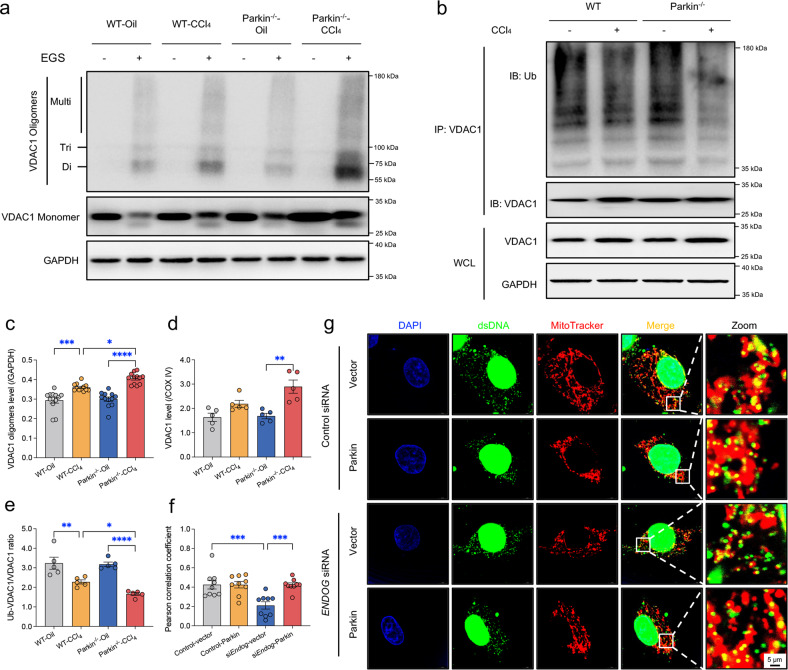
Effects of Parkin knockout on VDAC1 oligomerization and ubiquitination. **a** Representative immunoblots of VDAC1 monomer and oligomers in mouse livers. The crosslinking reagent EGS was used to stabilize the oligomers during electrophoresis. **b** Representative immunoblots of ubiquitinated VDAC1 in mouse livers. **c** Quantitative histogram of VDAC1 oligomers (*n* = 11-14/group). Quantitative histogram of (**d**) total VDAC1 in whole cell lysate (WCL) (GAPDH as the loading control) (*n* = 5/group) and (**e**) the ratio of ubiquitinated to total VDAC1 after IP with anti-VDAC1 antibody (*n* = 5/group). (**f**) Pearson correlation coefficient of dsDNA outside the nucleus and mitochondria (*n* = 9/group). siRNA targeting *ENDOG*, a nuclear-encoded mitochondrial endonuclease, was employed to induce mtDNA release in HepG2 cells. **g** Representative immunofluorescence images of cytosolic DNA accumulation in WT and *ENDOG*-knockdown HepG2 cells transfected or not transfected with Parkin using anti-dsDNA antibody (green) and MitoTracker (red). DAPI (blue) represents nuclear DNA, the yellow dots in the merged images represent mtDNA (dsDNA colocalized with MitoTracker), and the green dots in the merged images represent cytosolic DNA (dsDNA not colocalized with mitochondria or the nucleus). Mean ± SEM, *p < 0.05, **p < 0.01, ***p < 0.001 and **** *p* < 0.0001 between the indicated groups.

VDAC1 has been implicated in the formation of mitochondrial pores through homo-oligomerization to release mtDNA in response to pathological stimuli^14^. To this end, we examined the oligomerization state of VDAC1 by immunoblotting following treatment with the cross-linking reagent EGS to stabilize the oligomers during electrophoresis^14,31^. CCl_4_ challenge resulted in a higher level of VDAC1 oligomers in Parkin^-/-^ mice than in WT mice (Fig. 5a, c). Because VDAC1 can be ubiquitinated as a result of elevated Parkin E3 ligase activity, we examined whether ubiquitination is responsible for the regulation of VDAC1 oligomers. Our data suggested that Parkin ablation increased the total VDAC1 levels induced by CCl_4_ injection (Fig. 5a, d). Furthermore, although the protein level of Parkin was upregulated in response to CCl_4_ challenge (Fig. 2a), VDAC1 was less ubiquitinated in the livers after CCl_4_ insult, and a more pronounced decline was detected in Parkin^-/-^ mice (Fig. 5b, e).

To minimize potential confounding variables, mtDNA mutations are commonly used to induce mtDNA release^13,14^. We studied HepG2 cells transfected with siRNA targeting *ENDOG*, a nuclear-encoded mitochondrial endonuclease, which possess higher levels of cytosolic mtDNA and similar levels of total mtDNA relative to WT HepG2 cells^14^. As detected by immunofluorescence, the knockdown of *ENDOG* robustly increased the levels of cytosolic mtDNA in WT HepG2 cells but not in HepG2 cells overexpressing WT Parkin (Fig. 5f, g), suggesting that Parkin suppresses mtDNA release upon stress stimuli. These findings indicate that CCl_4_ challenge suppresses the E3 ligase activity of Parkin, leading to dampened ubiquitination of VDAC1, which may contribute to VDAC1 oligomerization.

### Parkin restricts mtDNA release through VDAC1 oligomers in hepatocytes

**Fig. 6:**
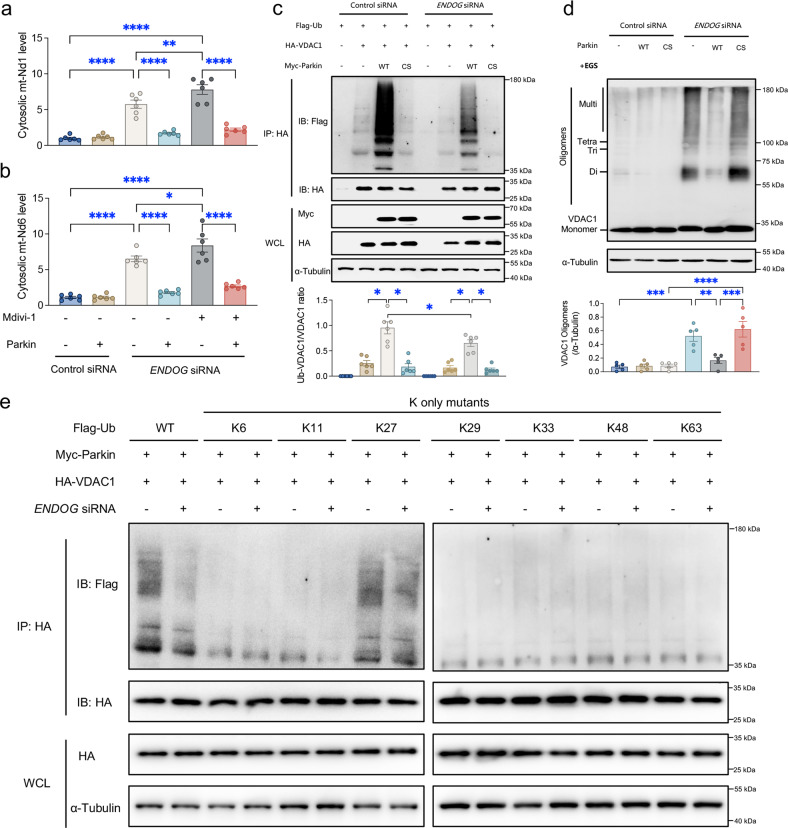
Effects of Parkin-mediated ubiquitination on VDAC1 oligomerization and cytosolic mtDNA accumulation in HepG2 cells. **a**, **b** Relative ratio of cytosolic to total mtDNA (mt-Nd1 and mt-Nd6) determined by qPCR analysis of WT and *ENDOG*-knockdown HepG2 cells expressing Parkin induced by treatment with the selective mitochondrial division/mitophagy inhibitor Mdivi-1 (50 μM, 24 h) (*n* = 6/group). Representative immunoblots and quantitative histogram of (**c**) the results from a ubiquitination assay with WT Parkin and E3 activity-loss Parkin C431S (CS Parkin) (*n* = 6/group) and (**d**) VDAC1 oligomers (α-Tubulin as the loading control) (*n* = 6/group). The positions of VDAC1 monomers (Monomer), dimers (Di), trimers (Tri), and multimers (Multi) are indicated. **e** Parkin ubiquitinated VDAC1 with Lys27 ubiquitin linkages. WT or *ENDOG*-knockdown HepG2 cells expressed Myc-Parkin, HA-VDAC1, and Flag-Ub WT or K-only mutants (K6, K11, K27, K29, K33, K48, and K63) are indicated (*n* = 3/group). Mean ± SEM, *p < 0.05, **p < 0.01, ***p < 0.001 and **** *p* < 0.0001 between the indicated groups.

Parkin mediates the regulation of mitochondria mainly by initiating mitophagy^12^. Hence, we examined whether the inhibition of mitophagy dampens Parkin-mediated hepatic protection against mtDNA release. Although Mdivi-1, a selective mitochondrial division/mitophagy inhibitor^33^, further increased the levels of cytosolic mtDNA in *ENDOG*-deficient HepG2 cells, it did not affect the protective effect of Parkin on dampening cytosolic mtDNA in the presence of *ENDOG* siRNA (Fig. 6a, b). To further explore the possible mechanism(s) through which Parkin regulates VDAC1, we monitored the ubiquitination of VDAC1 in the presence of WT Parkin or E3 activity-deficient Parkin C431S (CS Parkin) when *ENDOG* was knocked down. Notably, VDAC1 was polyubiquitinated in HepG2 cells expressing WT Parkin but not CS Parkin, indicating an indispensable role of the catalytic activity of Parkin in triggering VDAC1 polyubiquitination (Fig. 6c). *ENDOG* deficiency suppressed the ubiquitination of VDAC1 by WT Parkin (Fig. 6c). Moreover, the oligomerization of VDAC1 induced by *ENDOG* knockdown was impaired by WT Parkin but not by CS Parkin (Fig. 6d). We next used another set of ubiquitin mutants in which all but one lysine (K) residue was replaced by arginine (R). Although most ubiquitin K-only mutants could not significantly confer Parkin-mediated VDAC1 polyubiquitination, K27-ubiquitin promoted chain formation on VDAC1 as potently as WT ubiquitin (Fig. 6e). *ENDOG* knockdown apparently impaired Lys27 ubiquitin linkage in HepG2 cells (Fig. 6e). Collectively, VDAC1 oligomers seemingly restrict its ubiquitination by Parkin, whereas Parkin limits VDAC1 oligomerization in an E3 activity-dependent manner.

### Ubiquitination of VDAC1 at position 53 by Parkin impedes its oligomerization

**Fig. 7:**
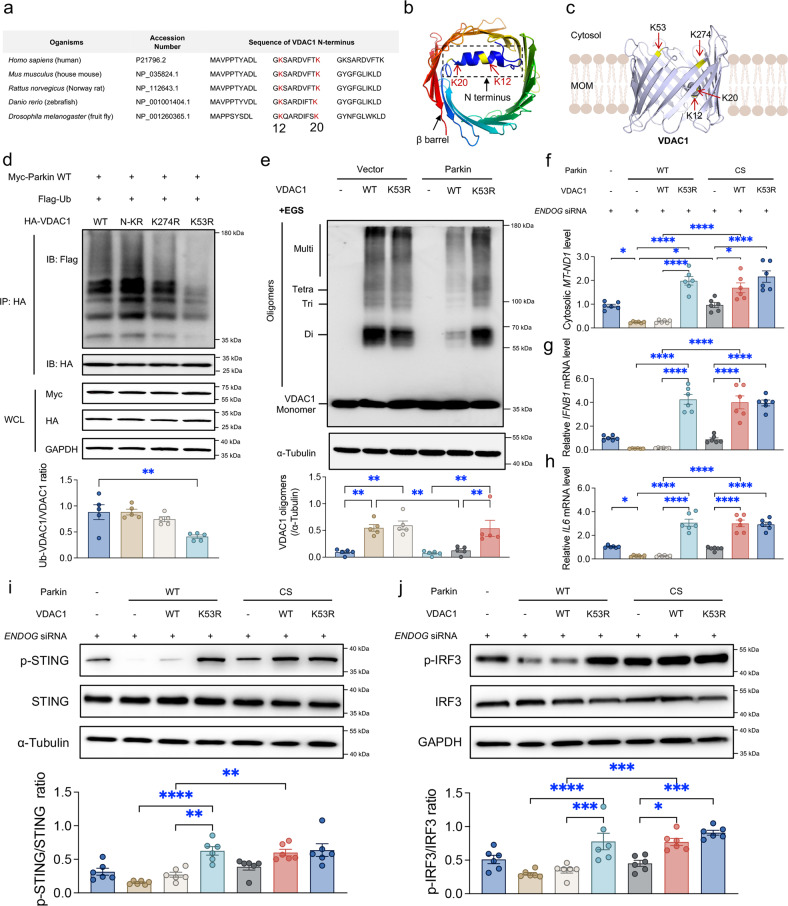
Identification of the VDAC1 ubiquitination site by Parkin. **a** Protein sequences of the VDAC1 N-terminal domain in 5 species. **b**, **c** Three-dimensional human VDAC1 protein structure (Protein Data Bank, ID code 2JK4) showing the indicated ubiquitination sites. Representative immunoblots and quantitative histogram of (**d**) the results from a ubiquitination assay with VDAC1 WT and its mutants bearing Lys-to-Arg substitutions (N-KR, K274R, and K53R) (*n* = 6/group) and (**e**) VDAC1 oligomers (α-Tubulin as the loading control) (*n* = 6/group). **f** Ratio of cytosolic to total mtDNA in *ENDOG*-knockdown HepG2 cells expressing WT/CS Parkin and WT/K53R VDAC1 as indicated. Relative mRNA or protein levels of (**g**) IFNB1, (**h**) IL6, (**i**) p-STING/STING, and (**j**) p-IRF3/IRF3 in LX-2 cells incubated with conditioned media from *ENDOG*-knockdown HepG2 cells expressing WT/CS Parkin and WT/K53R VDAC1 as indicated. Mean ± SEM, *p < 0.05, **p < 0.01, ***p < 0.001 and **** *p* < 0.0001 between the indicated groups.

Having established that the ubiquitination of VDAC1 blocks its oligomerization, we subsequently examined which Lys residues in VDAC1 might be the major ubiquitylation sites involved. The N-terminal domain of VDAC1 was shown to be obligatory for the transportation of mtDNA across the MOM when VDAC1 is in an oligomerized state^14,34^. We aligned the protein sequences of the VDAC1 N-terminal domain over various species and identified two evolutionarily conserved lysine residues (K12 and K20) that might be ubiquitinated (Fig. 7a, b), which are two of the three (K12, R15, and K20) positively charged sites in VDAC1 oligomers that bind mtDNA^14^. A quantitative proteomics analysis identified K53 and K274 in the cytosolic loops of the VDAC1 β-barrel (Fig. 7c) as the strongest candidates for Parkin-dependent ubiquitylation in human neurons^35^. Therefore, we substituted these indicated lysine residues (K) with arginine (R) to block ubiquitination at the mutated site (N-KR containing both K12R and K20R, K274R and K53R) and performed ubiquitination assays of HepG2 cells with these mutations. We found that VDAC1 polyubiquitination by WT Parkin was abolished in VDAC1 K53R cells, and monoubiquitination was slightly reduced in VDAC1 K274R (but not VDAC1 N-KR) cells (Fig. 7d).

We subsequently questioned whether the ubiquitination-defective VDAC1 K53R mutation interferes with the protective effect of Parkin on downstream signaling cascades. WT Parkin was found to limit the oligomerization of WT VDAC1 but not VDAC1 K53R (Fig. 7e). Moreover, WT Parkin reduced the cytosolic mtDNA release evoked by WT VDAC1 but not K53R VDAC1, whereas CS Parkin had little effect on either WT VDAC1 or K53R VDAC1 (Fig. 7f). Because STING is mainly expressed in stellate cells and/or Kupffer cells^2^, LX-2 human hepatic stellate cells were then cultured for 24 hours in conditioned media from HepG2 cells (Fig. 7g–j). VDAC1 K53R mutation endowed marked resistance to the Parkin-mediated response. HepG2 cells expressing VDAC1 K53R and WT Parkin evoked increases in ISG expression (Fig. 7g–h), p-STING levels (Fig. 7i), and p-IRF3 levels (Fig. 7j) in LX-2 cells than HepG2 cells expressing WT VDAC1 and WT Parkin. Together, our data highlight an essential role of Parkin-dependent ubiquitination of VDAC1 at K53 in restraining the formation of VDAC1 oligomers and subsequent innate immune activation.

## Discussion

**Fig. 8:**
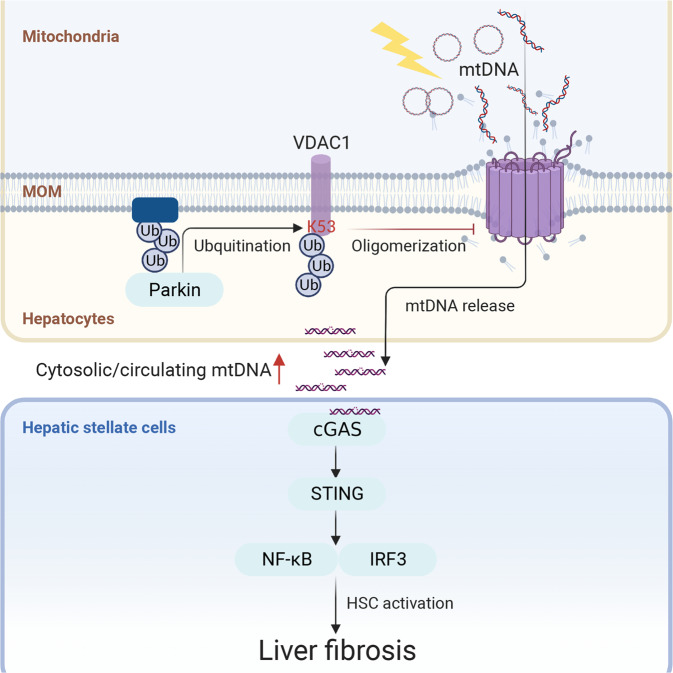
Proposed role of Parkin in the development of hepatic mtDNA release. Loss of Parkin-mediated ubiquitination of VDAC1 at position K53 and prevention of VDAC1 oligomerization augmented the release of mtDNA and the activation of STING signaling in liver fibrosis.

Parkin is a powerful regulator of mitochondrial integrity. In the present study, we employed mouse livers, human serum, liver tissues, and cell lines to validate the involvement of Parkin in the regulation of hepatic innate immune activation in liver fibrosis. Parkin knockout aggravates the autophagy suppression, cell death induction, and liver fibrosis induced by CCl_4_. Furthermore, Parkin is needed to prevent mtDNA release from injured mitochondria and subsequent activation of STING signaling evoked by CCl_4_ or *ENDOG* knockdown. Mechanistically, Parkin-mediated K27-linked ubiquitination of VDAC1, particularly on the K53 residue of VDAC1, markedly decreases VDAC1 oligomerization, mtDNA release, and activation of the STING signaling cascade. Collectively, our data have unveiled a unique role of Parkin in the regulation of mtDNA release through VDAC1 oligomerization and STING activation in liver fibrosis (Fig. 8).

Based on our data, suppression of autophagy, activation of STING signaling, and induction of cell death are reproducible responses to CCl_4_ insult in mice. CCl_4_ injection in close succession, as stress stimuli occur in human liver fibrosis^4^, results in mitochondria damage and cell death associated with the release of mitochondria-derived DAMPs, which triggers STING-dependent innate immune activation and progression of liver fibrosis. Previous studies have demonstrated that STING deficiency prevents cell death, inflammation, and fibrosis in various liver disease models, such as acute or chronic CCl_4_ injection^1^, diet-induced nonalcoholic fatty liver disease (NAFLD)^2^, and early alcoholic liver disease (ALD)^36^. Notably, markedly increased levels of cytosolic and circulating mtDNA were noted in our animals and human patients with liver fibrosis, in line with previous results observed in obese subjects with elevated ALT levels^9^. Therefore, these findings open up the possibility that serum mtDNA detection can be used for the diagnosis of liver disease.

To efficiently restrain innate immunity, a properly coordinated regulatory machinery is essential. Parkin has been implicated in limiting mitochondria-derived DAMPs in the face of mitochondrial damage^13,37^. Both acute and chronic mitochondrial stress may prompt increases in serum mtDNA and the STING-mediated response in mice lacking Parkin or PINK1^13^. However, the mechanism underlying the increased levels of cytosolic and circulating mtDNA in the absence of Parkin remains elusive. Deciphering how Parkin exert this effect is paramount to reconciling the mechanism through which the loss of Parkin promotes innate immune activation to evoke liver fibrosis. Our study revealed that Parkin directly modulates immune activation by suppressing the formation of VDAC1 oligomers to release mtDNA in an E3 activity-dependent manner. Parkin-mediated Lys27-linked polyubiquitinated VDAC1 was reluctant to form oligomers.

Parkin shows a preference for ubiquitylating lysine residues in the cytosolic β-barrel loops of VDAC1 because the hydrophilic α-helical N-terminus contains lysine residues that are not detectably targeted^35^ unless they are translocated out of the channel when VDAC1 is in an oligomerized state^14^. A previous study reported that Parkin is capable of inducing monoubiquitination on K274 and polyubiquitination on K12, 20, 53, 109, and 110 of VDAC1 to regulate apoptosis and mitophagy, respectively^37^. In our present study, we identified that VDAC1 polyubiquitination mainly occurs on K53 of the β-strand but not on K53 of the N-terminus. The ubiquitination-defective VDAC1 K53R mutation significantly nullified the Parkin-induced suppression of VDAC1 oligomerization. The cytosolic mtDNA release caused by VDAC1 K53R oligomers was not affected by Parkin. The main cell types involved downstream should be HSCs and/or Kupffer cells rather than hepatocytes because STING is rarely present in human hepatocytes^2^. Thus, we cultured LX-2 human HSCs in conditioned medium from HepG2 cells. Parkin inhibited WT VDAC1-induced mtDNA release and prevented subsequent activation of STING signaling in LX-2 cells. Based on these findings, we conclude that the ubiquitination of VDAC1 K53 by Parkin impairs the ability of VDAC1 to permit mtDNA release.

Although cytosolic mtDNA is inflammatory, it is also recognized by immune receptors other than STING, such as TLR9^9^ and NLR family pyrin domain containing 3 (NLRP3)^38^, leading to the similar cytokine profiles observed in liver disease. Thus, the relationship between mtDNA release and STING activation observed in our study may not be exclusive. In addition, because all types of cytosolic dsDNA activate STING^39^, we cannot exclude the possibility that nuclear DNA is involved. We speculate that mtDNA and nuclear DNA contribute to liver fibrosis depending on the severity of cell damage. Moreover, it has been reported that unstable amino acids in the β-strands 1, 2, and 19 of VDAC1 are needed for its oligomerization^40^. It may be possible that monoubiquitination on K274 of β-strand 19 disturbs VDAC1 oligomerization^37^. Therefore, how immune sensors differentially respond to dsDNA and how Parkin regulates dsDNA in the liver merit further investigation.

In conclusion, our current study offers mechanical insight into the pathogenesis of liver fibrosis. In particular, Parkin negatively modulates the ability of VDAC1 to oligomerize and release mtDNA in hepatocytes by specifically ubiquitinating K53 in VDAC1. The circulating levels of mtDNA may serve as a biomarker of liver disease. Our studies also suggest that designing molecules that promote the activity of Parkin and suppress the release of mtDNA may be desirable to inhibit the proinflammatory response in the liver.

## Supplementary information

Supplementary M&M

## Data Availability

The datasets used and/or analyzed supporting the findings of this study are available in this paper or the Supplementary Information. Any other raw data that support the findings of this study are available from the corresponding author upon reasonable request.

## References

[1] Iracheta-Vellve A (2016). Endoplasmic Reticulum Stress-induced Hepatocellular Death Pathways Mediate Liver Injury and Fibrosis via Stimulator of Interferon Genes. J. Biol. Chem..

[2] Luo X (2018). Expression of STING Is Increased in Liver Tissues From Patients With NAFLD and Promotes Macrophage-Mediated Hepatic Inflammation and Fibrosis in Mice. Gastroenterology.

[3] An P (2020). Hepatocyte mitochondria-derived danger signals directly activate hepatic stellate cells and drive progression of liver fibrosis. Nat. Commun..

[4] Brenner C, Galluzzi L, Kepp O, Kroemer G (2013). Decoding cell death signals in liver inflammation. J. Hepatol..

[5] Mills EL, Kelly B, O’Neill LAJ (2017). Mitochondria are the powerhouses of immunity. Nat. Immunol..

[6] West AP (2015). Mitochondrial DNA stress primes the antiviral innate immune response. Nature.

[7] Kang JW, Hong JM, Lee SM (2016). Melatonin enhances mitophagy and mitochondrial biogenesis in rats with carbon tetrachloride-induced liver fibrosis. J. Pineal Res..

[8] Zhao Y (2019). p66Shc Contributes to Liver Fibrosis through the Regulation of Mitochondrial Reactive Oxygen Species. Theranostics.

[9] Garcia-Martinez I (2016). Hepatocyte mitochondrial DNA drives nonalcoholic steatohepatitis by activation of TLR9. J. Clin. Invest.

[10] West AP, Shadel GS (2017). Mitochondrial DNA in innate immune responses and inflammatory pathology. Nat. Rev. Immunol..

[11] Deretic V (2021). Autophagy in inflammation, infection, and immunometabolism. Immunity.

[12] Wu NN, Zhang Y, Ren J (2019). Mitophagy, Mitochondrial Dynamics, and Homeostasis in Cardiovascular Aging. Oxid. Med. Cell. Longev..

[13] Sliter DA (2018). Parkin and PINK1 mitigate STING-induced inflammation. Nature.

[14] Kim J (2019). VDAC oligomers form mitochondrial pores to release mtDNA fragments and promote lupus-like disease. Science.

[15] McArthur K (2018). BAK/BAX macropores facilitate mitochondrial herniation and mtDNA efflux during apoptosis. Science.

[16] Bernardini JP (2019). Parkin inhibits BAK and BAX apoptotic function by distinct mechanisms during mitophagy. EMBO J..

[17] Saijou E (2018). Neutrophils alleviate fibrosis in the CCl(4)-induced mouse chronic liver injury model. Hepatol. Commun..

[18] Guo R, Xu X, Babcock SA, Zhang Y, Ren J (2015). Aldehyde dedydrogenase-2 plays a beneficial role in ameliorating chronic alcohol-induced hepatic steatosis and inflammation through regulation of autophagy. J. Hepatol..

[19] Ajaz S, Czajka A, Malik A (2015). Accurate measurement of circulating mitochondrial DNA content from human blood samples using real-time quantitative PCR. Methods Mol. Biol..

[20] Wang S (2021). Ablation of Akt2 and AMPKα2 rescues high fat diet-induced obesity and hepatic steatosis through Parkin-mediated mitophagy. Acta Pharm. Sin. B.

[21] Xu H (2021). TAX1BP1 protects against myocardial infarction-associated cardiac anomalies through inhibition of inflammasomes in a RNF34/MAVS/NLRP3-dependent manner. Sci. Bull..

[22] Ren J (2020). FUNDC1 interacts with FBXL2 to govern mitochondrial integrity and cardiac function through an IP3R3-dependent manner in obesity. Sci. Adv..

[23] Yang M (2021). Deletion of the E3 ubiquitin ligase, Parkin, exacerbates chronic alcohol intake-induced cardiomyopathy through an Ambra1-dependent mechanism. Br. J. Pharmacol..

[24] Hernandez-Gea V (2021). Co-expression gene network analysis reveals novel regulatory pathways involved in porto-sinusoidal vascular disease. J. Hepatol..

[25] Love MI, Huber W, Anders S (2014). Moderated estimation of fold change and dispersion for RNA-seq data with DESeq2. Genome Biol..

[26] Yu G, Wang LG, Han Y, He QY (2012). clusterProfiler: an R package for comparing biological themes among gene clusters. Omics.

[27] Schoggins JW (2014). Pan-viral specificity of IFN-induced genes reveals new roles for cGAS in innate immunity. Nature.

[28] Barcena C (2015). Angiogenin secretion from hepatoma cells activates hepatic stellate cells to amplify a self-sustained cycle promoting liver cancer. Sci. Rep..

[29] Yuan W-C (2014). K33-Linked Polyubiquitination of Coronin 7 by Cul3-KLHL20 Ubiquitin E3 Ligase Regulates Protein Trafficking. Mol. Cell.

[30] Wang S (2020). ALDH2 contributes to melatonin-induced protection against APP/PS1 mutation-prompted cardiac anomalies through cGAS-STING-TBK1-mediated regulation of mitophagy. Signal Transduct. Target Ther..

[31] Nagakannan P, Islam MI, Karimi-Abdolrezaee S, Eftekharpour E (2019). Inhibition of VDAC1 Protects Against Glutamate-Induced Oxytosis and Mitochondrial Fragmentation in Hippocampal HT22 Cells. Cell Mol. Neurobiol..

[32] Ajoolabady A, Aslkhodapasandhokmabad H, Aghanejad A, Zhang Y, Ren J (2020). Mitophagy Receptors and Mediators: Therapeutic Targets in the Management of Cardiovascular Ageing. Ageing Res. Rev..

[33] Lin XH (2020). Suppressing DRP1-mediated mitochondrial fission and mitophagy increases mitochondrial apoptosis of hepatocellular carcinoma cells in the setting of hypoxia. Oncogenesis.

[34] Abu-Hamad S (2009). The VDAC1 N-terminus is essential both for apoptosis and the protective effect of anti-apoptotic proteins. J. Cell Sci..

[35] Ordureau A (2018). Dynamics of PARKIN-Dependent Mitochondrial Ubiquitylation in Induced Neurons and Model Systems Revealed by Digital Snapshot Proteomics. Mol. Cell.

[36] Petrasek J (2013). STING-IRF3 pathway links endoplasmic reticulum stress with hepatocyte apoptosis in early alcoholic liver disease. Proc. Natl Acad. Sci. U. S. A..

[37] Ham SJ (2020). Decision between mitophagy and apoptosis by Parkin via VDAC1 ubiquitination. Proc. Natl Acad. Sci. U. S. A..

[38] Zhong Z (2018). New mitochondrial DNA synthesis enables NLRP3 inflammasome activation. Nature.

[39] Ablasser A, Hur S (2020). Regulation of cGAS- and RLR-mediated immunity to nucleic acids. Nat. Immunol..

[40] Geula S, Naveed H, Liang J, Shoshan-Barmatz V (2012). Structure-based analysis of VDAC1 protein: defining oligomer contact sites. J. Biol. Chem..

