# Supplementary material for: Site-specific ubiquitination of VDAC1 restricts its oligomerization and mitochondrial DNA release in liver fibrosis
Source: Exp Mol Med. 2023 Jan 19;55(1):269–80. doi: 10.1038/s12276-022-00923-9 (PMC9898252; doi:10.1038/s12276-022-00923-9)
Supplement: Supplementary file 1 — Supplementary M&M [file 12276_2022_923_MOESM1_ESM.pdf]

Supplementary Table 1: Patient information for liver tissues.

Variable	Normal (n=7)	Liver fibrosis (n=7)	*p* value
**Age (year)**	40.86 ± 2.68	43.00 ± 0.44	n.s.
**Gender (female/male)**	Female	Female	n.s.
**ALT (U/L)**	12.43 ± 0.95	31.86 ± 7.51	< 0.05
**AST (U/L)**	15.71 ± 0.94	34.14 ± 7.39	< 0.05
**Total bilirubin (μmol/L)**	11.63 ± 0.95	41.73 ± 29.68	n.s.
**Direct bilirubin (mol/L)**	3.66 ± 0.34	20.44 ± 17.84	n.s.
**Serum total protein (g/L)**	79.23 ± 1.58	65.60 ± 2.28	< 0.001
**Serum globulin (g/L)**	31.91 ± 0.90	29.50 ± 2.58	n.s.
**Serum albumin (g/L)**	47.31 ± 0.91	36.10 ± 2.39	< 0.001
**GGT (U/L)**	12.43 ± 0.69	93.43 ± 35.09	< 0.05
**ALP (U/L)**	56.57 ± 2.84	113.86 ± 41.94	n.s.
**PLT (*10^9/L)**	237.43 ± 25.30	201.14 ± 36.68	n.s.
**APRI**	0.18 ± 0.02	0.50 ± 0.14	< 0.05

Supplementary Table 2: Patient information for blood samples

Variable	Normal (n=12)	Liver fibrosis (n=12)	*p* value
**Age (year)**	41.25 ± 2.04	43.42 ± 0.53	n.s.
**Gender (female/male)**	Female	Female	n.s.
**ALT (U/L)**	12.58 ± 0.74	72.42 ± 19.95	< 0.01
**AST (U/L)**	15.67 ± 0.63	83.67 ± 19.87	< 0.01
**Total bilirubin (μmol/L)**	13.99 ± 1.45	76.88 ± 30.28	< 0.05
**Direct bilirubin (mol/L)**	4.38 ± 0.5	53.13 ± 24.01	n.s.
**Serum total protein (g/L)**	78.64 ± 1.16	61.25 ± 2.57	< 0.0001
**Serum globulin (g/L)**	32.24 ± 0.81	29.45 ± 1.85	n.s.
**Serum albumin (g/L)**	46.40 ± 0.78	33.47 ± 1.99	< 0.0001
**GGT (U/L)**	11.83 ± 0.58	106.17 ± 28.84	< 0.01
**ALP (U/L)**	56.00 ± 3.02	122.42 ± 26.57	n.s.
**PLT (*10^9/L)**	238.5 ± 17.46	175.83 ± 24.73	n.s.
**APRI**	0.17 ± 0.01	1.51 ± 0.41	< 0.01

ALT: alanine transaminase; AST: aspartate transaminase; GGT: Y-Glutamyl transpeptidase, ALP: alkaline phosphatase; PLT: Blood platelet; PLT: APRI: AST to platelet radio index = (AST/ULN*100)/PLT (*10E9/L), ULN = Upper limit of normal value of AST; n.s.: not significant.

Supplementary Table 3: Expression levels of interferon-stimulated genes (ISGs) in dataset GEO171248

Gene	HNL1	HNL2	HNL3	HNL4	HNL5	HNL6	HNL7	HNL8	LC1	LC2	LC3	LC4	LC5	LC6	LC7	LC8	log2FC	pvalue
CXCL10	20	25	46	34	179	53	211	26	190	22360	9120	4425	3964	4101	10108	1934	6.1227	1.47283E-17
LAMP3	2	2	14	6	9	5	15	0	161	1438	522	571	480	385	584	147	5.6200	2.83895E-33
CXCL11	4	27	3	9	19	1	20	1	12	1470	892	203	258	273	303	99	5.3426	1.00115E-12
ISG15	286	418	336	116	238	222	232	250	5071	40393	9222	10953	8777	4957	14253	4736	5.1403	3.34986E-42
ADAMDEC1	1	21	3	0	0	4	2	2	73	17	190	15	125	32	4	8	4.7777	4.42701E-10
OASL	164	273	107	81	158	160	163	82	779	13878	4111	2761	1970	2438	8587	1858	4.6845	1.51182E-29
IFI27	459	605	1036	448	537	308	494	382	1723	39400	19227	18401	22689	29080	40980	15906	4.6684	1.38431E-19
IFI6	651	551	837	343	486	351	335	448	10912	35057	20410	15445	15748	15621	23522	13036	4.5320	2.64525E-42
HSH2D	19	51	36	14	12	23	39	6	55	1400	541	598	661	457	761	352	4.5117	1.98753E-25
ETV7	3	5	26	11	9	8	13	7	105	833	262	328	137	238	388	124	4.3470	2.30463E-24
MX1	402	722	638	389	698	336	883	522	7301	32670	12536	11148	11583	9277	14146	5471	4.1137	7.06152E-45
ZBP1	5	0	9	11	13	6	9	0	19	584	151	105	103	60	179	41	4.0298	8.08997E-13
RSAD2	64	85	60	86	139	44	350	59	279	8310	1649	2139	1514	1172	2877	720	3.9055	1.90965E-11
CXCL9	76	74	53	155	408	84	193	34	73	1596	3279	1142	2734	6431	1492	1109	3.6383	7.14851E-09
IFI44L	271	346	317	382	391	82	936	157	981	13872	7578	4422	5649	5059	7616	1731	3.5205	3.83334E-16
OAS2	396	299	635	400	690	255	847	469	3447	15765	9609	6664	6451	5640	9622	3426	3.4969	4.90261E-34
OAS3	377	385	404	298	375	222	716	336	2229	12485	5503	5921	4264	3070	7667	2658	3.4414	2.53783E-29
IFI44	268	297	422	357	471	134	693	267	2132	12987	7593	4722	4526	3929	7033	2129	3.3998	4.43948E-23
CCL19	19	51	131	35	17	169	30	4	419	411	247	1036	2299	600	549	121	3.2872	5.29458E-07
ISG20	95	95	104	34	51	114	60	61	519	2931	831	780	777	405	898	255	3.2355	2.71957E-19
LGALS3	105	118	260	186	232	128	96	74	3634	1153	4880	1389	2155	831	477	524	3.1858	3.66213E-16
MX2	37	58	97	98	68	51	235	47	218	3239	1186	739	852	608	1012	445	3.1754	2.94334E-13
EPSTI1	136	85	150	111	243	73	484	188	886	5202	2941	2114	2123	1633	2315	891	3.0982	2.17103E-18
HERC6	129	123	214	143	103	104	292	185	969	4165	2450	1714	1706	1863	2403	706	3.0971	5.47993E-27
SERPINE1	40	60	291	187	117	367	206	107	9068	484	924	1898	1112	203	175	111	2.9998	0.000162711
IFIT1	1598	2230	691	1115	1154	1030	1515	876	3861	31779	15610	11456	8479	9993	15646	4424	2.9842	4.2503E-21
DDX60	242	175	303	269	354	214	676	306	1077	6329	4675	3917	2928	3012	4898	1288	2.9571	5.66476E-23
BCL2L14	5	0	11	16	16	7	11	8	4	273	133	84	55	65	108	18	2.7933	9.3283E-08
STAT1	1679	1788	1758	1287	2383	1190	3195	1678	6223	28842	24473	18079	15949	15252	17586	6918	2.7867	8.13584E-27
IFIT3	534	371	366	579	588	403	1289	521	1686	14758	6281	4691	3583	4652	6206	1846	2.7442	2.08459E-15
APOL3	730	929	664	469	723	485	857	1103	1653	10331	5517	7284	4358	3876	6817	3970	2.6905	4.46039E-20
HLA-F	461	621	875	433	780	540	395	373	1063	7601	4364	4136	3933	2953	4300	3137	2.5997	4.66028E-17
RTP4	96	56	120	94	130	72	128	131	224	2190	995	731	556	492	926	322	2.4773	4.90786E-14
IFIT2	652	869	368	1046	725	596	1239	554	1248	15815	6168	3960	3354	4493	5769	1548	2.4375	4.75338E-11
OAS1	947	1028	754	540	648	627	1031	716	2816	12108	5129	5610	4087	4085	6166	2630	2.4167	1.17037E-21
CCL8	0	0	8	3	4	2	26	0	2	141	97	10	8	30	11	15	2.3673	0.004593146
IFITM1	1027	1049	1713	534	989	1234	1475	583	4129	16132	6223	4186	4843	5634	9611	3766	2.3436	1.8263E-14
SLFN5	221	212	325	371	250	258	338	273	564	5081	2347	543	1071	2224	2508	562	2.2644	4.16623E-10
MAFF	12	15	57	34	17	74	32	9	298	240	347	171	216	49	162	79	2.1896	4.1427E-08
CLEC4E	1	21	6	0	12	1	4	4	19	15	91	13	21	25	10	3	2.1761	0.000513272
ARG2	43	44	21	4	3	42	42	14	408	26	86	13	518	50	53	15	2.1736	0.003332226
FUT4	42	76	72	33	38	33	77	32	307	226	581	181	351	279	184	84	2.1681	2.58643E-15
TYMP	618	519	1133	215	623	637	544	450	2930	7060	3455	3208	3177	2425	3190	1551	2.1655	7.18804E-15
FCGR1A	11	22	61	24	52	2	38	17	175	196	472	51	70	215	97	75	2.1521	1.13962E-05

**SOCS1**	**9**	**24**	**176**	**8**	**7**	**16**	**4**	**4**	**88**	**108**	**71**	**35**	**60**	**36**	**35**	**12**	**2.1471**	**6.49123E-08**
**LGALS9**	**168**	**309**	**443**	**220**	**352**	**147**	**198**	**197**	**1127**	**2222**	**2109**	**1074**	**1656**	**701**	**1149**	**487**	**2.0733**	**1.90629E-13**
**CD69**	**11**	**22**	**7**	**8**	**14**	**19**	**26**	**7**	**30**	**83**	**105**	**86**	**144**	**79**	**51**	**19**	**2.0565**	**1.62656E-09**
**MCOLN2**	**1**	**21**	**10**	**4**	**37**	**8**	**15**	**2**	**14**	**66**	**110**	**54**	**74**	**145**	**34**	**16**	**2.0551**	**0.000169909**
**GEM**	**15**	**8**	**35**	**29**	**26**	**125**	**11**	**20**	**257**	**61**	**206**	**185**	**438**	**256**	**122**	**31**	**2.0430**	**5.90118E-06**
**PSMB9**	**110**	**188**	**337**	**180**	**292**	**152**	**303**	**197**	**378**	**2115**	**1234**	**1186**	**988**	**1061**	**1625**	**760**	**2.0177**	**1.63916E-11**
UPP2	22	18	260	14	154	53	298	174	162	512	548	1461	873	374	1063	221	1.9793	0.002029326
GBP3	335	362	377	273	303	225	503	259	1303	1611	1501	1928	1702	1945	2315	907	1.9657	3.39592E-26
MAB21L2	5	0	21	36	1	28	23	2	52	163	137	102	97	42	107	29	1.9462	7.17234E-05
USP18	466	383	234	343	296	216	322	317	741	3958	1285	1333	1208	802	1529	802	1.9107	4.66013E-11
C15orf48	5	0	14	6	5	6	23	3	994	38	110	18	40	32	40	17	1.9000	9.11635E-05
CCL5	122	153	111	103	331	197	173	42	125	1147	1397	660	868	1012	570	279	1.8766	5.2399E-07
IFI16	438	463	695	350	477	265	563	200	1404	2954	3063	1461	2299	1750	1788	704	1.8409	1.69578E-12
DEFB1	994	962	346	547	277	909	939	1027	4065	2224	7059	2934	3321	2897	3383	1273	1.8363	1.93235E-12
SLC15A3	143	161	308	137	229	86	179	172	419	1900	960	697	547	608	877	473	1.8223	1.81477E-09
RGS1	9	24	13	44	65	7	22	13	69	110	191	47	177	131	92	38	1.8196	6.59917E-05
TLR7	5	0	8	20	30	8	25	8	34	29	136	37	139	73	36	30	1.7791	4.36253E-05
SECTM1	37	58	75	45	105	30	96	39	116	757	259	163	144	184	241	128	1.7627	6.84881E-06
EHD4	217	446	273	201	181	201	163	269	933	1341	1156	795	855	762	894	356	1.7395	7.29475E-23
BATF2	76	74	125	74	30	87	118	91	197	857	387	308	318	247	383	201	1.7324	7.42353E-09
TAP2	418	605	523	304	524	380	446	417	895	3135	2236	1511	1879	1621	1989	960	1.7312	3.44434E-17
NOD2	17	39	24	27	26	22	44	10	108	170	131	61	101	115	81	35	1.7289	2.8216E-09
TAP1	432	506	526	328	701	498	541	399	913	3761	2928	1510	2112	1602	2010	983	1.6733	7.68968E-13
CCL2	21	15	188	122	53	89	163	46	595	387	687	230	501	387	270	97	1.6535	1.60444E-05
TIMP1	3086	1103	8875	1630	971	1941	1142	846	10618	5418	13775	4638	6833	4506	6919	2981	1.6504	2.45589E-08
PARP12	455	425	553	384	316	476	405	572	1051	3580	2237	1742	1545	1459	2160	776	1.6499	2.02361E-14
UBE2L6	1030	1075	1787	935	1263	1097	1307	1368	3657	8729	4329	4698	3652	4437	6268	2588	1.6331	2.85265E-14
DDX58	403	290	321	493	421	251	666	290	916	3720	2126	1508	1522	1278	1694	626	1.6194	1.41718E-09
HK2	17	39	48	36	48	13	60	16	174	162	190	70	202	102	57	48	1.5612	2.03396E-05
STAP1	1	21	1	2	0	2	14	5	7	21	10	12	37	10	5	0	1.5469	0.051061429
IRF7	483	306	507	191	240	557	226	430	843	2978	1683	912	1033	1047	1807	618	1.5428	3.88578E-08
GZMB	3	5	11	10	5	11	36	1	7	51	64	49	106	18	35	17	1.5124	0.005082349
GBP1	654	677	651	757	901	738	1953	737	1253	7048	3261	2491	2381	3863	3820	1744	1.4892	8.62363E-07
CD74	5547	5148	11201	7326	15676	3573	8168	5061	13395	29253	27273	22918	30224	34507	35489	21639	1.4825	6.08268E-07
IFI30	672	877	1550	595	1262	512	759	976	2819	4255	6650	1664	2167	2325	2848	1895	1.4654	3.82563E-07
TDRD7	250	275	258	193	203	241	296	249	564	1550	1105	846	712	737	896	326	1.4532	5.43416E-14
XAF1	1310	1419	2547	1798	1659	1395	2417	1008	3045	11239	7977	4615	6011	6636	8019	2879	1.4498	7.21167E-09
IRF1	476	500	460	477	470	437	565	383	839	2862	1915	1513	1699	1258	1562	808	1.4363	5.66858E-13
HLA-C	8417	7124	8878	3277	4355	5679	4822	9022	8698	39121	24153	17753	19848	12955	24152	17787	1.4098	5.65906E-07
MICB	17	39	34	22	18	20	19	13	33	133	89	37	72	52	77	55	1.3771	9.96902E-06
CCND3	119	121	234	122	176	149	116	128	323	922	616	641	554	254	473	204	1.3694	2.99048E-08
PMAIP1	1	21	4	16	3	2	6	0	15	13	27	9	26	13	7	9	1.3353	0.015897054
PML	326	327	352	284	279	381	250	320	709	1912	1092	941	1083	779	1064	455	1.3224	3.97273E-13
PLSCR1	446	450	674	400	424	414	755	386	1364	2964	2496	1423	1684	1374	1767	589	1.3198	1.80335E-09
IFIT5	273	357	284	376	288	338	352	214	825	1875	1232	772	913	1040	998	317	1.3136	1.02487E-10
HES4	10	13	50	15	3	26	20	7	54	100	68	48	82	26	57	25	1.3108	0.002168636
DDIT4	335	362	203	277	430	348	172	417	2660	361	804	846	1038	515	612	433	1.2907	0.000135336
GBP4	250	275	318	280	768	289	1308	367	499	3256	1947	1081	1690	1820	1644	653	1.2874	0.000505837
NPAS2	194	203	200	245	113	259	85	183	652	124	1657	271	359	1062	347	94	1.2780	0.001896341
TMEM51	47	44	53	53	76	48	21	21	172	81	189	97	227	205	96	53	1.2698	1.01404E-05
TRIM21	272	357	309	244	222	266	264	316	608	1437	976	751	832	680	893	366	1.2643	1.2402E-13
SERPINB9	65	67	188	129	114	116	180	117	441	395	615	286	643	389	419	154	1.2585	5.87203E-09
GK	958	492	595	313	895	942	1030	655	5639	1627	2717	1179	3007	2449	1571	566	1.2508	4.09372E-05
TNFAIP3	338	309	319	348	337	335	401	247	785	1449	1566	877	1110	699	1102	346	1.2429	1.3776E-11
VAMP5	330	427	409	181	222	323	140	341	719	1474	990	787	842	503	729	422	1.2224	1.33791E-07
TAGAP	20	25	19	15	25	31	81	16	35	103	133	84	165	94	93	43	1.2207	0.000389353
ATF3	60	84	65	133	56	212	42	109	338	531	275	150	215	297	234	103	1.2162	0.000238552
BST2	2093	1124	3379	1669	2316	2414	1829	2593	1071	13586	10093	6967	7345	7188	8889	4386	1.2124	0.000125035
CCL4	25	27	45	69	114	28	68	35	94	158	341	69	151	306	171	58	1.2077	0.000619066
MARCKS	179	152	219	314	309	160	212	122	787	325	1629	349	975	663	378	186	1.1917	9.83478E-05
UNC93B1	266	252	366	247	205	232	234	177	350	1325	786	793	719	493	831	342	1.1765	4.12363E-07

TREX1	200	192	101	39	79	84	56	122	85	487	452	318	307	171	349	109	1.1738	0.000227988
BLVRA	57	59	127	78	153	56	114	68	346	263	449	150	339	279	212	98	1.1556	1.11145E-05
BUB1	1	21	12	2	39	10	10	25	35	25	67	27	47	36	20	31	1.1541	0.025740617
EIF2AK2	672	877	519	759	916	585	1133	676	1498	2943	3203	1959	2398	2541	2083	893	1.1471	1.9874E-10
SP110	212	167	301	326	337	223	335	273	623	1528	862	720	822	617	949	387	1.1398	1.74226E-08
GTPBP2	294	240	427	179	232	316	251	434	810	1385	1241	829	934	666	777	320	1.1271	2.68878E-08
GBP5	90	27	178	151	335	107	644	103	141	932	1070	315	704	1001	537	312	1.1243	0.007669674
B2M	32200	50262	62002	28164	51643	36458	37258	28111	84068	127177	140158	85114	119080	142330	115574	66420	1.0997	8.88837E-09
UBA7	799	721	818	686	923	865	639	803	979	3940	2015	2421	2196	1706	2778	1416	1.0888	7.12039E-07
CD80	2	2	10	11	10	0	3	2	6	29	33	5	33	9	23	5	1.0828	0.063820234
DUSP5	104	135	107	116	172	101	155	101	869	270	489	198	334	203	208	97	1.0823	0.000402315
PSMB8	1049	746	1140	612	973	1082	827	1007	1321	3988	2605	2596	2218	2100	3311	1646	1.0584	1.21609E-07
SNN	143	161	184	157	203	143	130	134	393	559	537	403	465	409	402	163	1.0521	4.8971E-13
IRF9	671	675	894	521	851	675	835	724	1390	2895	2096	1878	2041	1928	2472	1244	1.0483	6.33496E-10
TRIM14	890	844	1012	997	1092	595	652	1128	1497	3654	2668	2544	1865	2245	2586	1334	1.0272	2.17567E-07
CD274	62	86	57	86	44	57	79	56	64	336	198	105	175	156	187	58	1.0234	0.000261234
HPSE	20	25	49	52	119	22	28	29	106	116	283	59	101	142	85	40	1.0183	0.008561597
HLA-E	5100	6661	7131	5866	7940	5605	6509	3453	8201	21988	17979	13777	15974	16556	16257	10529	1.0154	5.24345E-07
MB21D1	13	35	23	24	28	14	10	15	38	48	43	37	104	37	43	15	1.0026	0.001233635
CNP	848	977	779	614	606	755	460	717	979	2974	1772	2227	1866	1109	1941	1176	0.9971	2.7437E-06
GMPR	129	123	96	44	118	104	78	88	87	540	272	242	238	149	308	110	0.9965	0.000602213
C4orf33	118	98	91	167	269	121	292	139	583	419	599	402	569	440	589	118	0.9940	8.28542E-05
APOL2	1670	865	868	782	774	1047	790	1000	1732	4622	2003	2577	1816	1648	2647	1445	0.9656	2.01977E-05
OPTN	1202	1161	951	1097	1817	1466	1335	1103	2720	4090	3115	3653	3799	2687	3242	1527	0.9589	7.57011E-11
BTN3A3	398	468	505	518	939	468	641	825	480	2087	1492	1207	1545	1675	1847	1169	0.9556	0.000293472
TRIM5	489	379	280	288	435	378	506	599	695	1559	1373	795	1088	898	1133	542	0.9392	3.35076E-07
GTPBP1	377	385	392	244	307	480	229	319	453	1655	796	826	775	508	848	477	0.9252	8.25848E-05
CCR1	53	125	108	162	168	41	185	71	210	357	556	107	240	242	230	174	0.9115	0.007543926
RASSF4	741	611	1707	498	616	847	539	437	1045	1680	2498	1734	2316	2386	2262	1017	0.8986	0.000481908
SAMHD1	627	677	828	688	1228	651	997	604	950	3033	2472	1592	2153	1893	1712	1032	0.8854	4.85563E-05
HEG1	299	386	410	345	404	340	305	278	1135	700	670	674	1139	756	789	301	0.8821	3.93013E-06
IL15	39	60	48	54	49	21	65	52	81	128	189	83	209	106	101	47	0.8749	0.000282342
ANKRD22	4	27	26	0	16	1	57	3	20	62	77	13	18	46	19	13	0.8333	0.23761467
TRIM38	448	422	512	474	624	486	627	559	797	1847	1444	892	1288	1293	1261	612	0.8265	6.26541E-07
PADI2	2	2	9	0	13	0	15	6	7	10	17	16	11	37	3	7	0.8239	0.22546661
IFITM3	10704	9256	13461	6278	9429	10841	7573	7144	24521	31516	18758	19844	16236	14689	25867	11685	0.8211	4.64127E-05
IL15RA	85	93	185	84	90	95	91	109	208	448	325	248	197	152	268	135	0.8204	0.00071004
DYNLT1	591	588	572	428	561	529	479	609	1294	2059	1534	1006	1298	1185	1171	485	0.8202	5.1044E-08
MSR1	171	151	248	246	448	44	307	136	437	502	1360	239	399	694	270	226	0.8182	0.031783896
DHX58	594	601	573	342	326	421	316	636	518	1639	1202	1056	921	823	1362	645	0.8180	0.000224019
THBD	137	104	103	83	153	103	61	100	382	211	178	317	281	99	228	68	0.8129	0.005948303
LAP3	3389	3496	3721	2807	3376	3206	5295	2665	5083	13258	7173	7740	6767	7599	9570	4116	0.8085	5.24813E-05
NMI	295	311	313	427	480	346	456	439	644	1539	1141	640	820	899	891	384	0.7974	3.17948E-05
ADAR	3103	4196	2964	2583	3227	2971	3120	3325	4460	9718	7705	6913	7231	6201	7046	3926	0.7965	1.27257E-09
FFAR2	7	2	4	19	0	5	0	3	6	21	22	5	12	11	9	8	0.7885	0.195748001
TRIM34	112	172	129	118	163	84	141	83	180	337	373	214	303	239	279	134	0.7872	2.24545E-05
ARHGEF3	59	46	162	126	243	94	133	71	278	232	378	219	474	258	281	98	0.7867	0.005139011
RARRES3	621	561	1267	513	571	694	658	779	536	3189	1624	1190	1062	1253	2444	1002	0.7849	0.008832872
TMEM140	1066	1481	955	954	855	962	1022	1032	1763	3223	2416	2136	1981	2197	2399	1189	0.7824	5.31809E-10
AIM2	6	0	23	16	52	2	11	10	0	32	79	32	50	63	15	22	0.7664	0.214946123
GCA	101	138	204	171	120	133	198	131	345	358	517	298	412	293	288	121	0.7647	7.43419E-05
TLR3	210	288	186	342	487	230	401	276	346	1017	758	700	659	629	762	283	0.7419	0.001533424
PNPT1	317	228	340	314	326	315	462	329	652	1280	949	609	742	633	725	287	0.7311	2.21104E-05
SPTLC2	277	227	349	293	332	254	320	314	832	688	969	545	717	629	537	237	0.7273	2.04204E-06
IFNGR1	928	710	1106	832	962	798	1158	768	2781	1806	3229	1532	2143	2134	1729	557	0.7152	0.000144199
FBXO6	271	346	170	118	141	179	122	238	207	620	407	292	264	395	356	258	0.7133	0.001915831
SPSB1	396	299	269	88	133	363	84	197	882	544	648	412	282	228	333	125	0.7076	0.041151894
VEGFC	45	101	75	46	54	39	15	10	121	47	102	55	116	122	71	28	0.7072	0.047207943
TRAFD1	307	398	387	254	306	350	367	388	417	1160	862	688	770	642	686	416	0.7065	4.499E-05
B4GALT5	562	694	436	374	509	477	299	496	1112	1226	1360	605	845	798	907	492	0.7012	1.31288E-05
LY6E	6654	4805	6661	3728	3213	5750	3525	2513	1687	15626	7972	10052	8626	6522	11855	8229	0.6907	0.035419439

PRKD2	281	322	367	269	328	350	247	248	398	969	592	749	805	485	617	306	0.6770	0.000136445
SIRPA	778	572	1421	821	442	1032	587	1055	2834	1264	2258	1244	2075	2071	1604	664	0.6770	0.005040732
TFEC	28	27	55	64	103	13	95	35	81	76	290	36	177	132	76	65	0.6599	0.098367478
SCO2	302	375	293	161	175	263	153	186	309	794	395	323	390	307	579	277	0.6335	0.005957287
DTX3L	1344	1062	1328	1430	1607	1354	1829	1729	2387	4838	3373	3035	2907	2968	3232	1357	0.6289	2.36857E-05
BLZF1	218	205	172	265	211	274	298	231	381	468	662	423	530	567	494	188	0.6242	7.43454E-06
LIPA	1627	1807	1864	2129	3837	1112	2088	1963	4381	3373	9202	3040	3040	4817	3472	1778	0.6234	0.014150634
CPT1A	3331	1555	2775	800	2245	4316	1916	2499	7984	5062	3433	3653	5044	4479	4891	2339	0.6204	0.019394543
N4BP1	589	479	454	555	497	539	478	517	723	1703	1143	941	1092	924	1034	494	0.6187	2.06518E-05
NDC80	4	27	14	12	25	14	15	38	27	40	44	22	51	26	21	23	0.6175	0.092797707
RNF24	49	78	67	68	74	80	75	64	143	118	226	138	199	107	125	33	0.6072	0.002273316
RBCK1	1541	1244	1178	898	975	1518	781	1629	1995	4228	2492	2206	1919	1607	2682	1235	0.6061	0.002724259
GBP2	479	542	857	643	762	537	935	607	1022	1850	2127	937	1604	1453	1249	544	0.6016	0.00199351
STAT2	4056	5470	4930	2900	3789	4367	3355	3937	5332	13492	7833	5959	7717	6390	9894	4113	0.5708	0.002263014
MYD88	1534	996	1443	1124	1258	1307	1273	1390	1737	4297	2195	2514	1885	2224	2711	1404	0.5693	0.002601087
APOL6	3174	3710	2529	3494	3806	3089	5501	3667	5002	10188	7851	6266	7185	6865	7880	3132	0.5617	0.000916441
RNF19B	171	151	184	189	148	169	183	134	246	501	381	311	313	255	284	175	0.5438	0.000814238
ETV6	285	251	304	217	248	257	310	312	551	615	647	338	695	466	449	239	0.5438	0.000260864
CD38	50	75	137	135	150	40	197	76	103	228	443	110	278	217	139	123	0.5347	0.120974177
TRIM25	2555	2151	2129	1348	1731	2605	2295	2662	3343	6526	4079	3261	4347	3625	4427	1925	0.5257	0.001307712
CASP7	345	392	406	322	443	420	395	390	527	1081	670	882	496	591	683	482	0.5248	0.005592019
MS4A4A	117	126	198	210	316	128	211	129	502	344	797	227	395	313	222	135	0.5222	0.052499015
CX3CL1	458	459	323	457	500	441	264	196	203	1084	896	755	796	508	807	458	0.5214	0.048839954
ANKFY1	880	521	624	906	820	662	901	829	939	1895	1697	1632	1517	1399	1563	817	0.5120	0.000284845
PHF11	336	258	442	323	399	301	411	385	906	822	910	472	878	575	720	269	0.5107	0.001221863
CYP1B1	199	250	67	188	105	91	150	132	513	131	276	175	428	297	121	82	0.4812	0.114668031
ATP10D	142	111	173	277	185	193	212	206	364	201	625	225	401	609	336	109	0.4644	0.048834724
C10orf10	632	526	3009	902	573	1466	1600	615	1456	2558	3389	1585	2505	2276	3908	882	0.4058	0.216765803
KIAA0040	210	288	387	221	187	272	297	181	353	498	524	457	578	348	403	225	0.4005	0.022937463
IL1RN	354	284	475	261	412	367	262	213	775	999	551	494	489	292	486	315	0.3957	0.098727707
PLEKHA4	435	492	588	394	238	416	280	198	294	1526	681	696	491	245	738	304	0.3953	0.217048317
WARS	671	675	737	503	1540	525	1452	957	953	2294	1929	1096	1545	1777	1346	825	0.3894	0.13371897
NAPA	2194	1717	1929	1354	1348	2115	1324	2117	2854	4134	2932	2887	3202	2136	3306	1473	0.3821	0.011704117
DCP1A	222	208	248	370	321	258	285	343	446	640	692	435	590	482	494	173	0.3794	0.013931173
SCARB2	5246	5106	3444	4947	5127	4084	5596	4445	7252	8876	9913	8955	8784	7990	7439	3392	0.3724	0.000729388
ALDH1A1	21742	27900	6990	15830	21642	22383	22462	18181	28355	31537	33630	29807	34757	44027	33119	16426	0.3698	0.04983273
PXK	196	233	176	232	178	217	236	182	239	504	445	352	347	307	337	138	0.3663	0.016597103
ADM	136	85	151	132	145	135	110	116	322	249	215	262	214	154	255	67	0.3663	0.050880493
NCF1	28	27	184	38	126	39	70	16	16	172	148	103	157	159	107	70	0.3226	0.478144787
ODC1	538	507	797	894	371	653	457	863	1663	807	1451	746	1273	1095	890	335	0.3216	0.167004691
ELF1	545	626	334	672	663	398	617	465	731	857	952	806	1118	911	846	379	0.3205	0.042693287
FAM46A	409	362	372	961	457	270	619	342	624	1486	1014	786	704	666	647	306	0.3113	0.243834886
MASTL	137	104	100	188	113	107	118	79	216	236	253	196	259	154	127	85	0.3065	0.106823947
FLT1	221	212	322	254	330	249	504	187	1013	215	392	470	733	511	345	126	0.3007	0.314463933
APOL1	3736	3162	3550	2799	4075	4275	4198	3943	4495	8284	5374	7569	4230	4218	7633	3360	0.3004	0.109034963
GPX2	1602	1269	8311	246	712	4814	523	3751	6791	2269	6490	3650	3073	4122	5631	1569	0.2827	0.628758292
ABTB2	518	773	254	252	467	372	283	398	393	768	412	638	559	441	561	429	0.2800	0.171403276
S100A8	26	25	33	38	37	71	85	31	71	67	145	37	77	73	84	14	0.2738	0.387635534
CHMP5	744	543	564	674	945	742	998	695	1366	1467	1415	1251	1256	1244	1225	545	0.2644	0.048565534
SSBP3	659	900	604	652	694	627	480	649	788	1010	762	869	745	1280	939	753	0.2538	0.145467598
PMM2	570	584	507	292	309	526	223	572	907	778	673	450	552	602	701	264	0.2510	0.25294036
FNDC3B	768	854	832	1402	572	635	812	874	1463	1077	2266	1015	1827	1431	1220	471	0.2464	0.215775057
PIM3	318	340	796	386	262	648	268	349	862	835	737	552	715	429	712	389	0.2408	0.321674444
AKT3	123	29	151	131	153	220	219	95	367	132	239	212	466	308	172	66	0.2396	0.355685121
MAX	394	354	474	510	554	526	541	510	644	1043	770	710	757	748	811	378	0.2169	0.096206964
CD163	1390	2131	3384	2640	3628	548	1971	1400	1802	3048	7711	1641	2018	4005	2532	2174	0.2151	0.546714539
PTMA	4506	3723	3639	3895	3655	4098	3798	3884	6954	6210	5716	5494	7369	5524	5668	2742	0.2147	0.015929294
FAM46C	177	143	332	217	129	200	317	299	395	442	408	302	422	272	440	150	0.2139	0.297520835
JAK2	95	95	96	177	205	108	316	114	155	321	361	183	298	265	284	76	0.2080	0.43947886
TXNIP	2404	3386	3450	8788	3571	6314	4740	3866	6176	4710	11061	8603	8066	7844	7102	1958	0.1989	0.440036239
IL17RB	1157	2144	896	976	871	1250	894	665	254	2384	2162	802	869	2157	2503	1132	0.1986	0.566583474

CLEC2B	62	86	112	180	242	95	202	59	220	230	300	92	307	248	188	74	0.1883	0.535955437
NCOA3	514	382	499	659	574	452	750	683	781	839	979	878	1197	994	792	376	0.1793	0.184289089
MAP3K14	233	278	376	185	200	349	189	228	212	520	291	401	506	278	431	230	0.1775	0.414057586
MCL1	4340	3287	3923	3345	4049	5229	3796	4159	6385	6794	9097	5227	5436	5019	6289	2786	0.1660	0.148427418
TLK2	318	340	241	320	407	302	361	364	442	603	563	427	511	551	452	218	0.1637	0.181824009
LGMN	1970	1767	1850	2058	1815	1397	1640	1100	2657	2920	3427	1674	2237	2431	2055	1559	0.1620	0.33330491
GAK	1386	1399	1168	913	972	1259	816	1269	1438	1879	1813	1579	1861	1402	1622	965	0.1592	0.154187039
PDK1	211	192	174	139	154	215	299	222	648	181	275	292	423	234	232	66	0.1537	0.572334376
LMO2	110	188	280	199	252	152	200	157	374	179	288	203	482	271	272	123	0.1405	0.543451017
PNRC1	611	684	980	754	1159	1032	1030	639	1176	935	2116	828	1496	1378	1323	564	0.1062	0.565316213
EXT1	1234	1125	1268	1153	724	1259	1102	1266	1824	1865	1700	1796	1371	1401	1465	925	0.1047	0.458391362
DDX3X	2534	3338	2889	3201	2274	2275	4054	2822	4973	2590	6677	3051	4107	5310	3856	1322	0.0988	0.618192615
GJA4	137	104	250	57	48	100	38	137	119	102	193	268	176	66	148	70	0.0979	0.786453689
GALNT2	3348	2809	3983	1001	1442	2310	1831	1869	3545	4055	2612	2689	2648	2451	3190	1736	0.0935	0.707099177
CREB3L3	2639	2189	5207	1622	1249	3714	2091	1744	4439	5663	5252	1968	1454	2368	4669	1670	0.0877	0.783734481
CCDC92	583	818	549	348	457	519	253	537	513	714	620	596	724	582	717	401	0.0872	0.585030901
LEPR	5472	7923	2177	2729	6155	4070	7231	2539	9113	7523	5816	3528	11722	3324	7435	2698	0.0775	0.78879973
PUS1	296	292	269	192	148	280	142	315	378	371	322	271	297	313	339	140	0.0741	0.679562107
PFKFB3	214	230	558	197	399	213	170	120	437	270	511	385	491	296	256	254	0.0690	0.808590119
NFIL3	370	501	360	578	387	806	412	365	652	391	926	239	626	1240	754	267	0.0647	0.807235246
CRY1	271	346	148	195	232	359	312	343	240	202	553	167	316	680	442	182	0.0061	0.981370612
IRF2	517	641	341	436	552	416	536	532	551	741	642	567	647	526	637	362	-0.0126	0.91956274
C4orf32	224	322	173	108	133	280	198	99	325	218	299	181	194	184	246	53	-0.0136	0.956160956
TCF7L2	442	503	300	390	530	300	381	415	480	562	557	357	572	572	512	236	-0.0666	0.622000362
ANGPTL1	180	222	114	227	287	533	1045	253	63	430	612	391	1120	637	628	248	-0.0805	0.852057458
IFITM2	3637	4965	6699	1951	2404	3347	2048	2497	7589	5212	3820	3447	3169	1360	2627	1908	-0.0899	0.779697571
GLRX	1835	1804	1483	1145	1207	1395	1572	1672	2680	2146	1963	1136	1238	1672	1883	940	-0.1028	0.562786728
EPAS1	3665	3163	3720	3451	3311	3771	5067	3236	5884	3694	4228	4847	5345	4249	4216	1999	-0.1120	0.438551759
STEAP4	554	511	1504	937	955	502	667	349	1076	881	1255	1039	730	1045	693	349	-0.1393	0.619668331
STARD5	796	707	1057	416	810	830	674	1397	336	1963	621	834	756	1237	1274	488	-0.1495	0.618974164
SMAD3	681	604	795	726	814	837	881	711	1035	1125	970	781	1262	787	1003	468	-0.1532	0.216676804
ULK4	33	34	32	54	25	36	23	37	69	27	75	25	64	46	29	6	-0.1583	0.610219296
NUP50	834	737	1329	911	856	911	1099	872	1478	978	1433	881	1452	966	1075	486	-0.1670	0.299232177
IL6ST	4466	6336	3663	4782	5150	3716	6909	3440	4334	5082	8208	6221	6897	6288	4747	2200	-0.1741	0.298132707
NRN1	38	48	58	50	57	53	135	61	155	37	66	65	78	54	92	590	-0.2153	0.487694223
TNFSF10	4069	7118	2545	7098	4467	2855	5871	3388	1269	10489	4892	4917	4412	3916	5649	3500	-0.2291	0.469873888
PI4K2B	1278	1095	542	921	1259	824	1382	1035	1081	1262	1175	1007	1340	1497	1119	537	-0.2362	0.147346101
CFB	117950	86935	143498	63316	95636	116548	86744	66264	119909	123225	121245	97091	77631	76795	117444	60706	-0.2770	0.148643685
FNDC4	1421	1315	1262	550	636	1218	452	838	1480	1037	1314	814	688	805	883	411	-0.2788	0.240138216
SLC25A30	1216	822	649	1025	1328	1471	964	944	1006	1198	1241	1189	906	1465	1160	513	-0.2828	0.084775221
BAG1	1190	962	589	587	637	817	707	820	543	1037	662	880	933	824	1008	431	-0.2990	0.063684651
PDGFRL	44	59	75	43	65	50	32	28	134	45	48	47	38	78	22	7	-0.3168	0.40093388
FKBP5	2599	775	1783	879	2487	1626	583	2599	1790	2215	1971	3895	1111	461	1181	719	-0.3192	0.392688261
ABLIM3	1772	1415	2999	2816	1163	3640	1463	3710	4903	2174	3835	2224	1802	2311	2395	801	-0.3428	0.210126528
ERLIN1	2518	2133	2554	1816	2386	2747	3491	1920	2285	2415	2365	2228	2902	2892	2515	1464	-0.3555	0.013755975
GCH1	1732	1358	863	936	1990	1087	1916	1459	2465	1251	1517	880	1594	1590	1313	543	-0.3709	0.093527922
COMMD3	870	1027	733	667	674	837	673	736	944	784	985	558	854	781	799	316	-0.3825	0.000611373
SLC1A1	640	682	2077	738	1192	1411	1739	1235	2142	784	1775	1254	1678	1335	943	368	-0.3951	0.14080313
PABPC4	1453	1427	1597	2221	1637	1759	1599	1358	1834	1682	1972	1574	1805	1625	1470	701	-0.4002	0.002030624
RNASE4	4756	3427	3746	2448	3433	5086	2894	5868	3766	3644	5882	3436	3583	3526	3533	2085	-0.4186	0.011148287
CD9	739	693	395	540	384	786	614	405	849	527	526	569	641	510	387	215	-0.4356	0.020333874
SOCS2	276	214	239	342	154	497	405	187	314	394	237	396	747	235	258	52	-0.4748	0.144911898
SLC16A1	1935	1782	765	1647	783	2496	2791	1005	1676	2024	1659	1772	1441	2513	1642	683	-0.4754	0.086390573
SERPING1	71380	45976	85069	64799	60853	83793	51109	53036	64753	62535	57722	53349	50544	51079	68081	40533	-0.4944	0.004371478
RBM25	2455	3200	1925	2992	2363	2208	2845	2650	2536	2509	2692	1913	3079	2555	2341	983	-0.5020	1.87646E-05
BCL3	461	621	1379	265	488	846	275	614	850	609	507	359	544	423	692	334	-0.5112	0.093174308
RAB27A	527	646	542	489	482	610	842	520	694	443	561	495	654	512	517	237	-0.5215	0.00027277
G6PC	4905	9003	5087	14547	11945	9984	12629	4978	9006	3748	8438	3338	8436	17955	6828	5083	-0.5448	0.092561194
MAP3K5	205	127	542	241	523	580	625	359	543	274	264	184	518	557	394	192	-0.5602	0.04740893
CEBPD	617	623	1383	884	661	739	417	371	595	855	653	536	486	483	1045	347	-0.5635	0.033501417
ARNTL	270	245	297	769	350	697	299	309	208	94	891	141	325	1030	357	152	-0.5844	0.14456534

RPL22	6066	6976	4128	7207	5347	5530	5628	4207	7200	3874	5216	4388	6822	5181	4257	1655	-0.5864	0.000511322
C1S	114761	91790	94090	95568	109039	109872	93959	84511	67296	87265	71500	84682	64260	99463	84832	51382	-0.6692	1.52382E-06
MAFB	746	826	504	1734	852	584	610	641	474	650	752	611	579	490	628	553	-0.6923	0.00753531
FAM134B	341	362	246	513	453	593	817	348	1390	55	595	227	447	323	325	78	-0.7265	0.102799679
CES1	74275	58223	43632	37811	52220	108821	58056	88844	18254	53440	30804	50191	42351	67489	55625	40857	-0.7715	0.00416939
PPM1K	316	357	208	483	664	314	612	519	324	364	388	245	600	443	379	99	-0.8132	0.001017395
HESX1	15	8	26	53	29	16	48	24	21	25	40	8	18	8	20	25	-0.8174	0.038501283
TBX3	1227	1077	959	1068	1847	1323	1344	1475	805	1052	782	832	1080	1082	1159	602	-0.8832	2.31695E-08
AMPH	25	27	18	14	7	7	6	8	6	5	10	7	20	3	7	7	-0.9140	0.035734392
ZNF385B	421	749	103	620	704	241	475	495	79	367	357	223	370	299	338	297	-0.9166	0.00803197
SAMD4A	939	1158	450	1108	716	702	1024	1152	490	710	808	583	524	556	617	352	-0.9547	1.07253E-07
ENPP1	3698	3865	1947	3445	3582	2538	4332	3077	1486	2092	1962	1919	2436	2263	1853	1181	-1.1144	3.38305E-13
AHNAK2	5	0	332	12	2	155	39	30	151	24	43	30	48	0	36	16	-1.1543	0.227118871
CRP	6852	2803	322756	1459	3498	77985	22102	17897	188670	403	41614	353	14318	16311	297	584	-1.1901	0.207083454
IGFBP2	13402	10053	10439	29009	4426	7881	5066	2961	1287	14698	2427	7939	1841	1789	5772	5892	-1.2052	0.053958672
MKX	22	18	13	15	15	37	35	18	9	14	20	21	14	4	10	2	-1.3073	0.0002891
IMPA2	426	486	1007	229	362	862	325	435	232	296	203	224	232	263	218	121	-1.4842	5.7171E-08
SAA1	24251	1014	463287	2029	1057	19670	2098	8265	24018	1038	11461	794	1367	648	1859	1636	-1.6710	0.021541699
**MT1M**	**487**	**438**	**1377**	**187**	**599**	**504**	**202**	**2977**	**448**	**346**	**63**	**154**	**138**	**495**	**77**	**229**	**-2.0015**	**0.001340833**
**MT1F**	**1036**	**1071**	**3094**	**752**	**1602**	**1585**	**513**	**7181**	**393**	**650**	**138**	**223**	**394**	**1570**	**233**	**503**	**-2.2522**	**0.000200374**
**MT1X**	**3929**	**3265**	**10517**	**2361**	**6260**	**6257**	**3039**	**15153**	**1863**	**1528**	**497**	**1310**	**2279**	**1980**	**873**	**1652**	**-2.3415**	**1.24799E-09**
**MTHFD2L**	**372**	**370**	**1031**	**268**	**315**	**864**	**560**	**569**	**214**	**153**	**156**	**119**	**136**	**77**	**131**	**60**	**-2.4358**	**3.03283E-18**
**MT1G**	**6496**	**3612**	**15941**	**8558**	**7725**	**8126**	**2115**	**38858**	**3603**	**3159**	**307**	**676**	**1923**	**7237**	**1133**	**2013**	**-2.4570**	**0.000277297**
**MT1H**	**765**	**654**	**3928**	**1274**	**855**	**538**	**149**	**6576**	**245**	**225**	**20**	**13**	**87**	**652**	**25**	**81**	**-3.7561**	**6.95552E-06**

Supplementary Table 4: Primers for RT-qPCR and genotyping

Species	Target	Forward Primer (5' to 3')	Reverse Primer (5' to 3')
**Human**	*MT-ND1*	CACTTTCCACACAGACATCA	TGGTTAGGCTGGTGTTAGGG
	MT-ND6	CCAATCCTACCTCCATCGCT	GAGTATCCTGAGGCATGGGG
	MT-ATP6	ACCACAAGGCACACCTACAC	TATTGCTAGGGTGGCGCTTC
	MT-RNR2	ATTGACCTGCCCGTGAAGAG	GCATGTACTGCTCGGAGGTT
	B2M	TGTTCCTGCTGGGTAGCTCT	CCTCCATGATGCTGCTTACA
**Mouse**	Genotyping	AAATGCCCAGACAAGATGCC	GGCCTCTCACGAVTGAGTT
	mt-Nd1	TCCGAGCATCTTATCCACGC	GTATGGTGGTACTCCCGCT
	mt-Nd6	TTAGCATTAAAGCCTTCACC	TAACAATCACCCAAACAACC
	Col1a1	GCTCCTCTTAGGGGCCACT	CCACGTCTCACCATTGGGG
	Acta2	GTCCCAGACATCAGGGAGTAA	TCGGATACTTCAGCGTCAGGA
	Mmp2	CAAGTTCCCCGGCGATGTC	TTCTGGTCAAGGTCACCTGTC
	Gapdh	AGGTCGGTGTGAACGGATTTG	TGTAGACCATGTAGTTGAGGTCA

MT-ND1: NADH dehydrogenase 1; *MT-ND6*: NADH dehydrogenase 6; *MT-ATP6*: mitochondrially encoded ATP synthase 6; *MT-RNR2*: mitochondrially encoded 16S RNA; *B2M*:

beta-2 microglobulin; *Col1a1*: collagen type I alpha 1; *Acta2*: actin alpha 2, smooth muscle; *Mmp2*: matrix metallopeptidase 2; *Gapdh*: glyceraldehyde-3-phosphate dehydrogenase

Supplementary Table 5: Antibodies used in immunoblotting.

**Target**	**Catalog**	**Company**
Autophagy related gene 5 (ATG5)	#109490	Abcam, Cambridge, England
Autophagy related gene 7 (ATG7)	#52472	
Sequestosome-1 (p62)	#91526	
Microtubule-associated proteins 1A/1B light chain 3B II (LC3B)	#192890	
Bcl-2-associated X protein (Bax)	#32503	
Apoptosis regulator Bcl-2 (Bcl2)	#17509	
Caspase-3	#184787	
Cytochrome C	#4272	
Voltage-dependent anion-selective channel 1 (VDAC1)	#154856	
Transcription factor p65 (NFκB p65)	#32536	
Phospho-NFκB p65	#76302	
Interferon beta (IFN-β)	#218229	
Tumor necrosis factor alpha (TNF-α)	#215188	
Parkin	#4211	Cell Signaling Technology, Danvers, MA, USA
Stimulator of interferon genes protein (STING)	#13647	
Phospho-STING (Ser366)	#19781	
Interferon regulatory factor 3 (IRF3)	#4302	
Phospho-IRF3 (Ser396)	#4947	
Interleukin (IL-6)	#12912	
GAPDH	#60004-1-Ig	Proteintech, Hubei, China
α-Tubulin	#11224-1-AP	
Cytochrome c oxidase subunit 4 (COX IV)	#11242-1-AP	
